# Gold(I) N-heterocyclic carbene precursors for focused electron beam-induced deposition

**DOI:** 10.3762/bjnano.12.21

**Published:** 2021-03-17

**Authors:** Cristiano Glessi, Aya Mahgoub, Cornelis W Hagen, Mats Tilset

**Affiliations:** 1 Department of Chemistry and Centre for Materials Science and Nanotechnology (SMN), Faculty of Mathematics and Natural Sciences, University of Oslo, P.O. Box 1126 Blindern, NO-0318 Oslo, Norway; 2 Delft University of Technology, Fac. Applied Sciences, Dept. Imaging Physics, Lorentzweg 1, 2628CJ Delft, Netherlands

**Keywords:** Au(I) precursors, focused electron beam-induced deposition (FEBID), gold-NHC, gold precursors, nanofabrication, N-heterocyclic carbene

## Abstract

Seven gold(I) N-heterocyclic carbene (NHC) complexes were synthesized, characterized, and identified as suitable precursors for focused electron beam-induced deposition (FEBID). Several variations on the core Au(NHC)X moiety were introduced, that is, variations of the NHC ring (imidazole or triazole), of the alkyl N-substituents (Me, Et, or iPr), and of the ancillary ligand X (Cl, Br, I, or CF_3_). The seven complexes were tested as FEBID precursors in an on-substrate custom setup. The effect of the substitutions on deposit composition and growth rate indicates that the most suitable organic ligand for the gold precursor is triazole-based, with the best deposit composition of 15 atom % gold, while the most suitable anionic ligand is the trifluoromethyl group, leading to a growth rate of 1 × 10^−2^ nm^3^/e^−^.

## Introduction

Focused electron beam-induced deposition (FEBID) is a nanofabrication technique that allows for the growth of three-dimensional free-standing nanostructures [1–4]. This mask-less nanofabrication technique uses gaseous molecules as precursors. The gas molecules are introduced in the specimen chamber of a scanning electron microscope (SEM), adsorb onto a substrate, and dissociate upon electron irradiation, leaving a solid deposit on the substrate and some volatile fragments. The technique has been employed in applications such as the fabrication of nanoconnectors [5], extreme ultra-violet lithography (EUVL) mask repair [6], AFM probe tips [7–9], nanodevices for plasmonics [10], gas sensors [11–12], optoelectronics [13], and magnetic [14–15] and biomedical applications [16]. FEBID provides a flexible direct-write technique to fabricate complex 3D structures, which are hard to realize using resist-based planar lithography processes. However, when using organometallic precursors, usually, undesired dissociation fragments also end up in the deposit. A major challenge is therefore to achieve control over the composition of the deposited material through a proper design of the precursor molecule [17–18].

**Figure 1:**
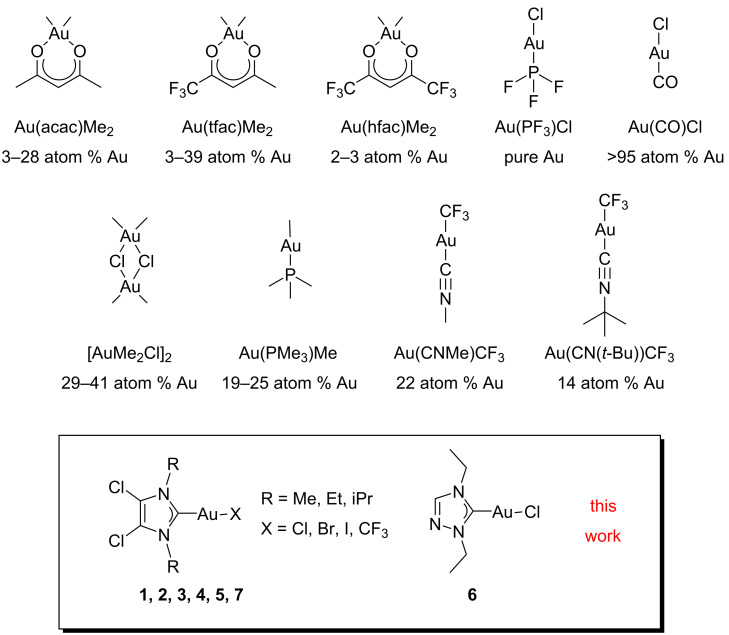
Overview of previously reported FEBID gold precursors, Au(acac)Me_2_ [10,20–22], Au(tfac)Me_2_ [21,23–25], Au(hfac)Me_2_ [19], Au(PF_3_)Cl [27–30], Au(CO)Cl [31], [AuMe_2_Cl]_2_ [32], Au(PMe_3_)Me [32–33], Au(CNMe)CF_3_ [34], and Au(CN(*t*-Bu))CF_3_ [34], and the compounds studied in this work, labelled as **1**–**7**.

Gold deposition has been one of the earliest interests in FEBID [19], as gold 3D-nanostructures can find a wide range of applications from plasmonics [10] to optoelectronics [13]. Gold FEBID precursors (Figure 1) have had a similar history as other metal precursors, as the first tested compounds were taken from the existing library of gold precursors for chemical vapour deposition (CVD). The first compounds tested were gold dimethyl acetylacetonate, Au(acac)Me_2_, and its trifluorinated and hexafluorinated derivatives, Au(tfac)Me_2_ and Au(hfac)Me_2_ [19]. While for the former two compounds the gold content in the deposits varied over a large range (3–28 atom % [10,20–22] and 3–39 atom % [21,23–25]), the latter complex yielded only traces of gold (2–3 atom % [19]). Within the series of gold acetonate complexes, the highest gold content has been achieved with Au(tfac)Me_2_ when water was co-injected as an oxidizing agent during the deposition (91 atom % gold) [26]. To circumvent carbon contamination, a series of inorganic gold(I) complexes has been explored, such as Au(PF_3_)Cl [27–30] and Au(CO)Cl [31], which gave high-purity deposits. Unfortunately, the high instability of these precursor molecules has severely hindered their use as FEBID precursors.

For the compounds [AuMe_2_Cl]_2_ and Au(PMe_3_)Me [32], it was demonstrated that the presence of alkyl ligands in gold FEBID precursors has a highly positive effect on the stability of the compounds [33–34] and can lead to a satisfactory purity of the obtained nanostructures (19–25 and 29–41 atom % Au, respectively) [32]. The most recent organometallic gold complexes that were tested are Au(CNMe)CF_3_ and Au(CN(*t*-Bu))CF_3_. These complexes are stabilized by the presence of a good σ-donor ligand (isocyanide) and their volatility is enhanced by the presence of a trifluoromethyl ligand. Deposits from these precursors contained 22 and 14 atom % of gold, respectively [34].

Although many different ligand architectures of gold organometallic complexes were tested as FEBID gold precursors, the effect of different substitutions in the core structure of the molecule on the composition and growth rate of deposits is still largely unexplored. Such studies may reveal groups or ligands in precursors that perform better in FEBID and can perhaps lead to a generalized precursor design.

The objective of this work is to expand the already existing library of gold(I) FEBID precursors and to study the effect of various substitutions at the core structure of a series of gold(I) N-heterocyclic carbene (NHC) complexes on the growth rate and composition of deposits. The precursors that were synthesized had the general formula Au(NHC)X, and the effect of the variation of both the NHC ligand and the ancillary ligand X (X = Cl, Br, I, CF_3_) (Figure 1) was studied. Because the sublimation temperatures of these precursors exceeded the maximum operating temperature of standard gas injection systems (GIS) in the SEM, an unconventional method was chosen to introduce the precursors from a custom-built reservoir mounted directly on top of a heated substrate on which also the deposits were directly grown. The growth of the deposited pillars was studied as a function of the deposition time, and some larger cube-shaped deposits were made to determine the composition of the deposited material using energy-dispersive X-ray (EDX) spectrometry.

## Experimental

### Synthesis and characterization

All chemicals and solvents needed were commercially available and were used as received. Au(SMe_2_)Cl was prepared according to a procedure reported in [35]. 1,4-Diethyl-1,2,4-triazolium iodide was prepared according to the procedure reported in [36]. In order to more easily refer to the complexes, we denote the compounds as (Y,R)AuX, where Y is the backbone substitution of the NHC ligand, R is the N-substituent, and X is the negatively charged ancillary ligand. The precursors (Cl,Me)AuCl (**1**), (Cl,Et)AuCl (**2**) and (Cl,iPr)AuCl (**3**) were prepared according to [37]. All reactions were performed under ambient conditions unless specified otherwise. NMR spectra were recorded on Bruker Advance DPX200, DPX300, AVII400, AVIII400 and AVII600 instruments at ambient temperature. ^1^H and ^13^C NMR spectra were referenced relative to the residual solvent system (CD_2_Cl_2_). ^19^F NMR spectra were referenced to hexafluorobenzene (−164.9 ppm). Mass spectra were obtained on a Micromass QTOF II spectrometer and a Bruker Daltronics maXis II spectrometer. Melting point determinations were performed on a Stuart SMP10 melting point apparatus, using flame-sealed capillaries at a pressure of ca. 0.2 mbar in order to mimic a vacuum environment.

[(Cl,Et)AuBr] (**4**): A solution of **2** (100.6 mg, 0.24 mmol, 1 equiv) and LiBr (211.6 mg, 2.4 mmol, 10 equiv) in dried acetone (10 mL) was stirred in the dark under Ar for 20 h. Solvent was removed by rotary evapouration and the resulting white solid was partially dissolved in dichloromethane (DCM) and purified through column chromatography (DCM, silica). The product was obtained as an off-white powder (100.3 mg, 89%). ^1^H NMR (400 MHz, CD_2_Cl_2_) δ 4.29 (q, *J* = 7.3 Hz, 4H, -C*H*_2_-), 1.44 (t, *J* = 7.3 Hz, 6H, -C*H*_3_); ^13^C NMR (101 MHz, CD_2_Cl_2_) δ 174.1 (NHC-*C*), 116.9 (=*C*-Cl), 46.3 (-*C*H_2_-), 16.0 (-*C*H_3_); MS (ESI^+^, MeOH): *m*/*z* 490.896 ([M(^35^Cl^35^Cl^79^Br) + Na]^+^, 62.3%), 492.894 ([M(^37^Cl^35^Cl^79^Br) + Na]^+^, [M(^35^Cl^35^Cl^81^Br) + Na]^+^, 100%), 494.891 ([M(^37^Cl^37^Cl^79^Br) + Na]^+^, [M(^37^Cl^35^Cl^81^Br) + Na]^+^, 46.6%); HRMS (MeOH): *m*/*z* meas. 490.8963, calcd. 490.8962 for [C_7_H_10_Au^79^Br^35^Cl_2_N_2_Na]^+^ (Δ = −0.1 ppm); mp 204–205 °C; elemental analysis: calcd. for C_7_H_10_AuBrCl_2_N_2_: C, 17.89; H, 2.14; N, 5.96; found: C, 17.76; H, 2.13; N, 5.82%.

[(Cl,Et)AuI] (**5**): A suspension of **2** (100.3 mg, 0.24 mmol, 1 equiv) and NaI (358.2 mg, 2.4 mmol, 10 equiv) in dried acetone (10 mL) was stirred in the dark under Ar for 20 h. Solvent was removed by rotary evapouration and the resulting white solid was partially dissolved in dichloromethane (DCM), filtered, and purified through column chromatography (DCM, silica). The obtained orange powder was precipitated from layering of DCM and pentane. The product was obtained as a white powder (89.2 mg, 72%). ^1^H NMR (600 MHz, CD_2_Cl_2_) δ 4.30 (q, *J* = 7.3 Hz, 4H, C*H*_2_-), 1.45 (t, *J* = 7.2 Hz, 6H, -C*H*_3_); ^13^C NMR (151 MHz, CD_2_Cl_2_) δ 181.0 (NHC-*C*), 117.0 (=*C*-Cl), 46.0 (-*C*H_2_-), 16.0 (-*C*H_3_); MS (ESI^+^, MeOH): *m*/*z* 538.882 ([M(^35^Cl^35^Cl) + Na]^+^, 100%), 540.880 ([M(^37^Cl^35^Cl) + Na]^+^ 64.5%), 581.010 ([(NHC)_2_Au]^+^, 74.1%), 583.007 ([(NHC)_2_Au]^+^, 95.9%), 585.005 ([(NHC)_2_Au]^+^, 46.3%); HRMS (MeOH): *m*/*z* meas. 538.8824, calcd. 538.8824 for [C_7_H_10_Au^35^Cl_2_IN_2_Na]^+^ (Δ = −0.1 ppm); mp 178–179 °C; elemental analysis: calcd. for C_7_H_10_AuC_2_lIN_2_: C, 16.26; H, 1.95; N, 5.42; found: C, 16.07; H, 1.91; N, 5.28%.

[(N,Et)AuCl] (**6**): 1,4-Diethyl-1,2,4-triazolium iodide (400.6 mg, 1.58 mmol, 1 equiv) was solubilized in 60 mL of DCM. Ag_2_O (185 mg, 0.80 mmol, 0.5 equiv) was added and the resulting suspension was left stirring in the dark for 15 h. To the resulting white suspension solid Au(SMe_2_)Cl (467.3 mg, 1.58 mmol, 1 equiv) was added and immediately a yellow coloration of the suspension was observed. After 4 h of stirring in the dark the yellow suspension was filtered and concentrated to dryness. The solid was purified by column chromatography (silica, DCM) and an orange product was obtained. Upon recrystallization from layered DCM and pentane, white crystals were obtained (565 mg, 84%). ^1^H NMR (400 MHz, CD_2_Cl_2_) δ 8.05 (s, 1H, =C*H*-), 4.41 (q, *J* = 7.3 Hz, 2H, -C*H*_2_-, N side), 4.24 (q, *J* = 7.4 Hz, 2H, -C*H*_2_-, CH side), 1.53 (t, *J* = 7.4 Hz, 3H, -C*H*_3_, CH side), 1.51 (t, *J* = 7.3 Hz, 3H, -C*H*_3_, N side); ^13^C NMR (101 MHz, CD_2_Cl_2_) δ 173.2 (NHC-*C*), 142.2 (=*C*H-), 49.2 (-*C*H_2_-, N side), 44.9 (-*C*H_2_-, CH side), 16.6 (-*C*H_3_, CH side), 15.7 (-*C*H_3_, N side); MS (ESI^−^, MeOH): *m*/*z* 356.023 ([M(^35^Cl)-H]^−^, 100%), 358.020 ([M(^37^Cl)-H]^−^ 31.9%), 392.000 ([M(^35^Cl)+Cl]^−^ 40.3%); HRMS (MeOH): *m*/*z* meas. 356.0233, calcd. 356.0234 for [C_6_H_10_Au^35^ClN_3_]^−^ (Δ = 0.3 ppm); mp 131–132 °C; elemental analysis: calcd. for C_6_H_11_AuClN_3_: C, 20.15; H, 3.10; N, 11.75; found: C, 20.09; H, 3.13; N, 11.78%.

[(Cl,Et)AuCF_3_] (**7**): AgF (104.0 mg, 0.82 mmol, 2 equiv) was added in a Schlenk flask. Upon addition of dry acetonitrile (10 mL) a grey suspension was obtained under vigorous stirring. Me_3_SiCF_3_ (0.3 mL, 2.1 mmol, 5 equiv) was added and a white/grey suspension was immediately formed. After a few minutes, the white suspension turned grey. The reaction mixture was stirred in the dark for 1 h. Solid **2** (176 mg, 0.41 mmol, 1 equiv) was added and the mixture turned into a light grey suspension. After 1 day the reaction mixture was concentrated to dryness and the obtained solid was partially dissolved in DCM, filtered, and concentrated. The dark product was purified by column chromatography (DCM, silica) and the product was obtained as a white powder (122.1 mg, 66%). ^1^H NMR (400 MHz, CD_2_Cl_2_) δ 4.28 (q, *J* = 7.3 Hz, 4H, -C*H*_2_-), 1.45 (t, *J* = 7.3 Hz, 6H, -C*H*_3_); ^13^C NMR (101 MHz, CD_2_Cl_2_) δ 184.5 (q, *J* = 14.9 Hz, NHC-*C*), 162.8 (q, *J* = 344.2 Hz, -*C*F_3_), 117.4 (=*C*-Cl), 46.1 (-*C*H_2_-), 16.5 (-*C*H_3_); ^19^F NMR (188 MHz, CD_2_Cl_2_) δ −30.61 (s, 3F, -C*F*_3_); MS (ESI^+^, MeOH): *m*/*z* 480.973 ([M(^35^Cl^35^Cl) + Na]^+^ 100%), 482.970 ([M(^37^Cl^35^Cl) + Na]^+^ 63.4%); HRMS (MeOH): *m*/*z* meas. 480.9730, calcd. 480.9731 for [C_8_H_10_Au^35^Cl_2_F_3_N_2_Na]^+^ (Δ = 0.2 ppm); mp 146–149 °C; elemental analysis: calcd. for C_8_H_10_AuCl_2_F_3_N_2_: C, 20.93; H, 2.20; N, 6.10; found: C, 20.99; H, 2.17; N, 6.05%.

### Determination of the sublimation temperature

Sublimation temperatures for compounds **1**–**7** were obtained by cold finger sublimation. The cold finger sublimation setup was immersed in an oil bath and heated by a heating plate. The temperature was controlled and registered by an immersion thermometer immersed in the oil bath at the same height as the bulk material and connected to the heating plate. The pressure measured for each experiment was 1 × 10^−3^ mbar on a VACUU.VIEW or DCP 3000 Vacuubrand manometer mounted on the Schlenk line used. The cold finger was cooled down with continuous water flow. 20 ± 1.5 mg of bulk material were charged in the sublimation apparatus, which was then evacuated and conditioned with Ar or N_2_ before being evacuated again. The dynamic heating of the sample was carried out at a heating rate of 1 K per 10 min. The sublimation temperature was determined as the point at which the formation of a white film was observed on the initially transparent cold finger. The material was left subliming at this temperature in order to accumulate a small quantity of sublimate, sufficient for further analysis. The sublimed material was then collected and analyzed by ^1^H NMR spectroscopy. The identity of the sublimed material was assessed by comparison with the ^1^H NMR spectrum of the bulk material (see Supporting Information File 1).

### Deposition setup

**Figure 2:**
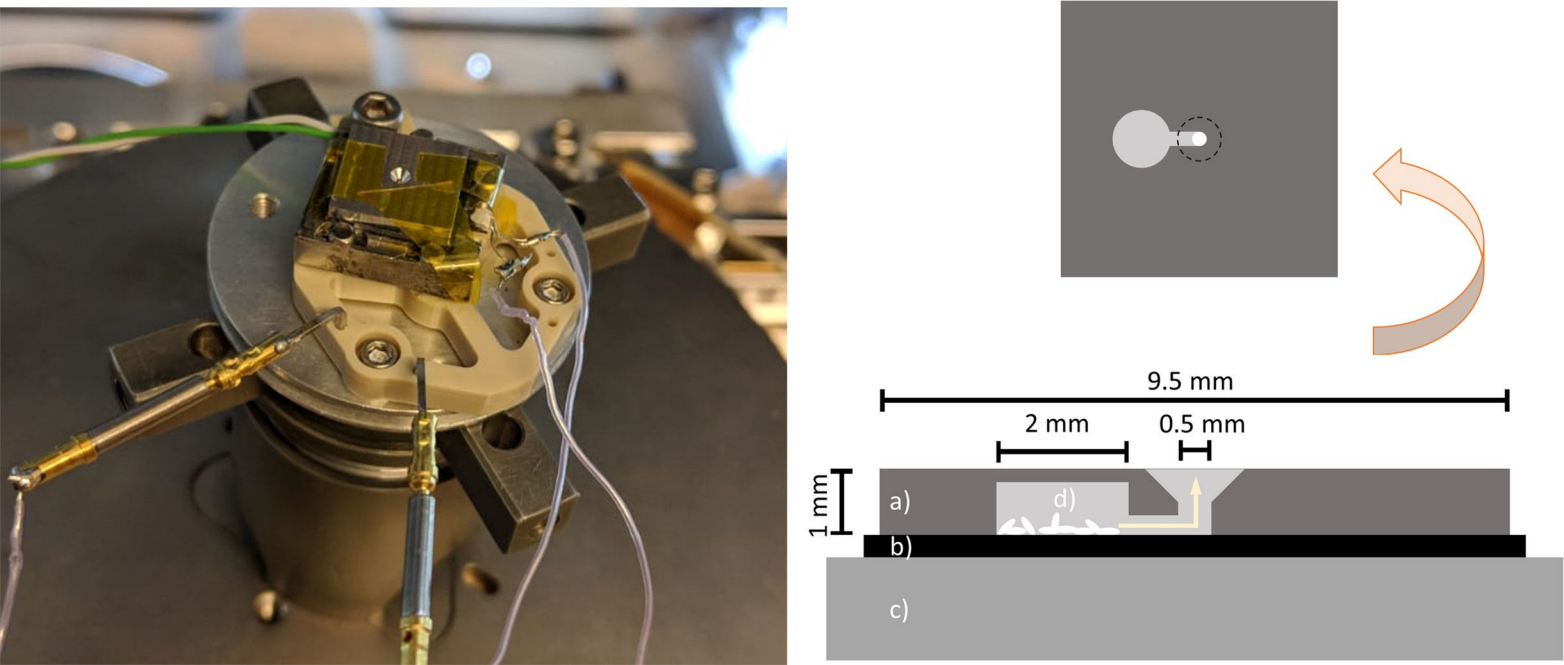
Substrate heater and precursor supply system of the deposition setup. Only the conical hole in the centre, through which the deposition is done, is visible. The precursor crystals are stored underneath the top metal cover. (a) Vapour guide, (b) silicon substrate, (c) substrate heater, and (d) precursor crystals. The sketch on top shows the bottom view of the vapour guide.

All deposition experiments were performed in a Thermo Fisher Scientific Nova Nanolab 600 dual-beam SEM. The base chamber pressure was about 1 × 10^−6^ mbar. Silicon substrates were used for all experiments. The silicon substrates were cleaned by ultrasonication in acetone for 15 min, followed by ultrasonication in isopropanol for 15 min and blow drying with N_2_, and were kept in a dust free environment. For each precursor a different substrate was used to avoid cross contamination. The substrate was mounted on a custom-built heater [38] shown in Figure 2 and crystals of the precursor were placed directly on the substrate. The low volatility of the compounds required heating above the maximum allowable temperature of a standard GIS to effectively sublime the material. An aluminium plate covered the substrate, leaving a central circular area free for deposition. The precursor material was contained in a recessed hole in the bottom of the plate, connected by a small channel to the deposition area. For each precursor to be tested a new plate was taken to avoid cross contamination.

Precursors were tested for deposition upon e-beam irradiation. When deposition was successful, two types of deposits were created. Firstly, large deposits for composition analysis were written by repeatedly (2000 passes) exposing a 250 × 250 nm^2^ area, using point exposures with a dwell time of 500 µs and a pitch of 10 nm between the exposure points. Secondly, to characterize the growth, square arrays of 3 × 3 pillars, each pillar grown at a different dwell time, were fabricated. In each array, the pillar separation was 1 µm. Two types of arrays were deposited, one with short dwell times of 0.1, 0.2, 0.5, 1, 2, 5, 10, 20, and 50 s (referred to as type 1), and one with longer dwell times for slow-growth precursors of 1, 5, 10, 20, 40, 60, 80, 100, and 120 s (referred to as type 2).

The Nova Nanolab has a 12-bit DAC to control the beam position. Therefore, the addressable grid runs from 0 to 4095 pixels in the *X* direction and from 280 to 3816 pixels in the *Y* direction. The horizontal field width (4.1 µm) is equivalent to 4096 pixels, that is, one pixel corresponds to 1 nm.

Dimensions of deposits were measured from SEM images, and, in case of tilt images, corrected for the tilt angle. For the determination of the deposited volume, pillars were approximated either as a cone or a cylinder with a cone on top, depending on their shape. The error was calculated based on an error of ±10 nm in measuring the height and diameters of the pillars, except for a few pillars where the height error was ±100 nm. These few pillars were much longer and were thus measured at a lower magnification than the other pillars. Beam energy and current used for all deposits were 5 keV and 40 pA, respectively.

### Composition determination

EDX measurements were performed in the same Nova Nanolab 600 dual-beam SEM using an Oxford Instruments X-MAX 80 EDX detector. Beam energy and current used during the measurements were 5 keV and 600 pA, respectively. The working distance was kept around 5 mm to have the optimum EDX signal. All spectra were analysed using the Oxford Instruments AZtec software.

## Results and Discussion

### Precursor design

The properties of a FEBID precursor molecule are crucial for the deposition process. The precursor molecule should be volatile in a suitable range of pressures and temperatures. Under these conditions, it needs to be easily deliverable in the gas phase, adsorb on a substrate, be sensitive to the electron beam, and decompose in a clean manner to the desired products. Furthermore, it should be inexpensive and easy to prepare, non-toxic, and easy to store and handle [17]. The choice of such molecules requires a compromise to be made between volatility, stability, and reactivity induced by electron irradiation of the molecule. This must then be translated into a structure that can lead to the deposition of the desired material [33]. Most FEBID precursors are organometallic complexes designed with the aim to obtain pure metal deposits. Normally, the metal content is quite limited, and the deposits are often heavily contaminated with carbon. Therefore, it makes sense to use as little carbon as possible in the design of the molecule [17]. Furthermore, it has been observed that large ligands, such as the methylcyclopentadienyl group in MeCpPtMe_3_ and acetylacetonate in Au(acac)Me_2_, do not decompose favourably under electron irradiation. However, this trend is not extended to the recently reported silver carboxylates [39–40]. In gold(I) complexes, only two ligands are present in the coordination sphere, a neutral ligand L and an anionic ligand X. Both ligands influence the complex stability, with an increased stability after the introduction of Au–C bonds [33]. The X ligand has shown to control the volatility of the complex by means of steric hindrance; as the size of X increases, the intramolecular interactions between the precursor molecules, specifically the aurophilic interactions, decrease substantially, rendering the precursor more volatile. This is valid only if the compounds maintain a constant lattice [33–34,41].

Gold(I) NHC complexes are known for their versatility in different applications such as catalysis [42], biomedicine [43], and photochemistry [44]. While chemically very different species are classified as NHCs, they all share a common moiety, a carbene carbon stabilized by two α-nitrogen atoms. NHCs are neutral two-electron donors, analogous to the more extensively studied CO and PR_3_ derivatives. The coordinative capability of the NHCs depends in fact primarily on the sp^2^-hybridized lone pair of the carbene carbon atom, which has a strong σ-donor capability [45]. Moreover, the presence of back donation of π electrons into the empty p*_z_* orbital of the carbene carbon atom further strengthens the C–Au bond [45]. Such features hint at a strong organometallic bond that precludes ligand dissociation under the temperature and pressure conditions involved in FEBID experiments.

**Figure 3:**
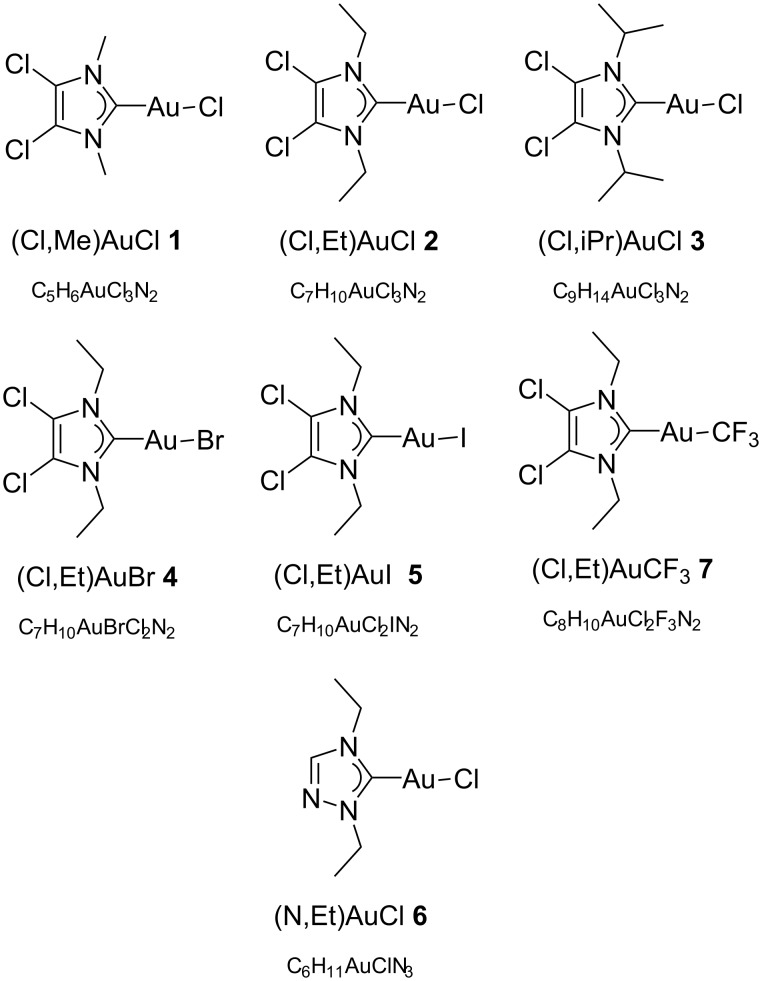
The selected Au(NHC)X complexes studied and their molecular formulae.

Gold(I) NHC complexes owe their widely ranged adaptability to the great variability of the NHC ligand itself [46]. The NHC ligand can, in principle, be tailored to the desired application. For FEBID the main aim is to diminish the number of carbons in the molecular formula as much as possible in order to minimize the tendency to form contaminated deposits. Furthermore, we aim at the introduction of various heteroatoms such as halogens and nitrogen, in an effort to increase the volatility of the compound. These criteria were fulfilled in the NHC gold(I) complexes **1**–**7** (Figure 3). Compounds **1**–**5** and **7** are imidazole-based NHC complexes with short aliphatic substituents on the nitrogen atoms (N-substituents) and two chlorine atoms on the backbone of the aromatic structure. While compounds **1**–**3** have a chloride as the other ancillary ligand, this has been modified in compounds **4**, **5**, and **7** with the introduction of a bromide, iodide, or trifluoromethyl group, respectively. The effect of the halogen substitution on the performance of FEBID precursors has, to the best of our knowledge, not been investigated yet for gold compounds. The difference between the bromide and chloride ligands has been investigated [47] for platinum precursors. It was shown that under the tested conditions, the chloro compound performed better than the bromo compound in terms of composition and growth rate [47].

Compound **6** is a triazole-based NHC gold(I) complex that presents a further reduced number of carbon atoms compared to the imidazole-based compounds with the replacement of one of the backbone carbon atoms by a nitrogen atom.

Based on the design of these precursors, the expectation is that electron irradiation will decompose the ligands under the formation of nitrogen- and chlorine-containing organic fragments, hopefully leading to more volatile by-products, compared to precursors with purely carbon-based ligands. Furthermore, the synthesis aimed for a series of thermally robust compounds that can be straightforwardly handled and tested.

#### Synthesis

**Scheme 1:**
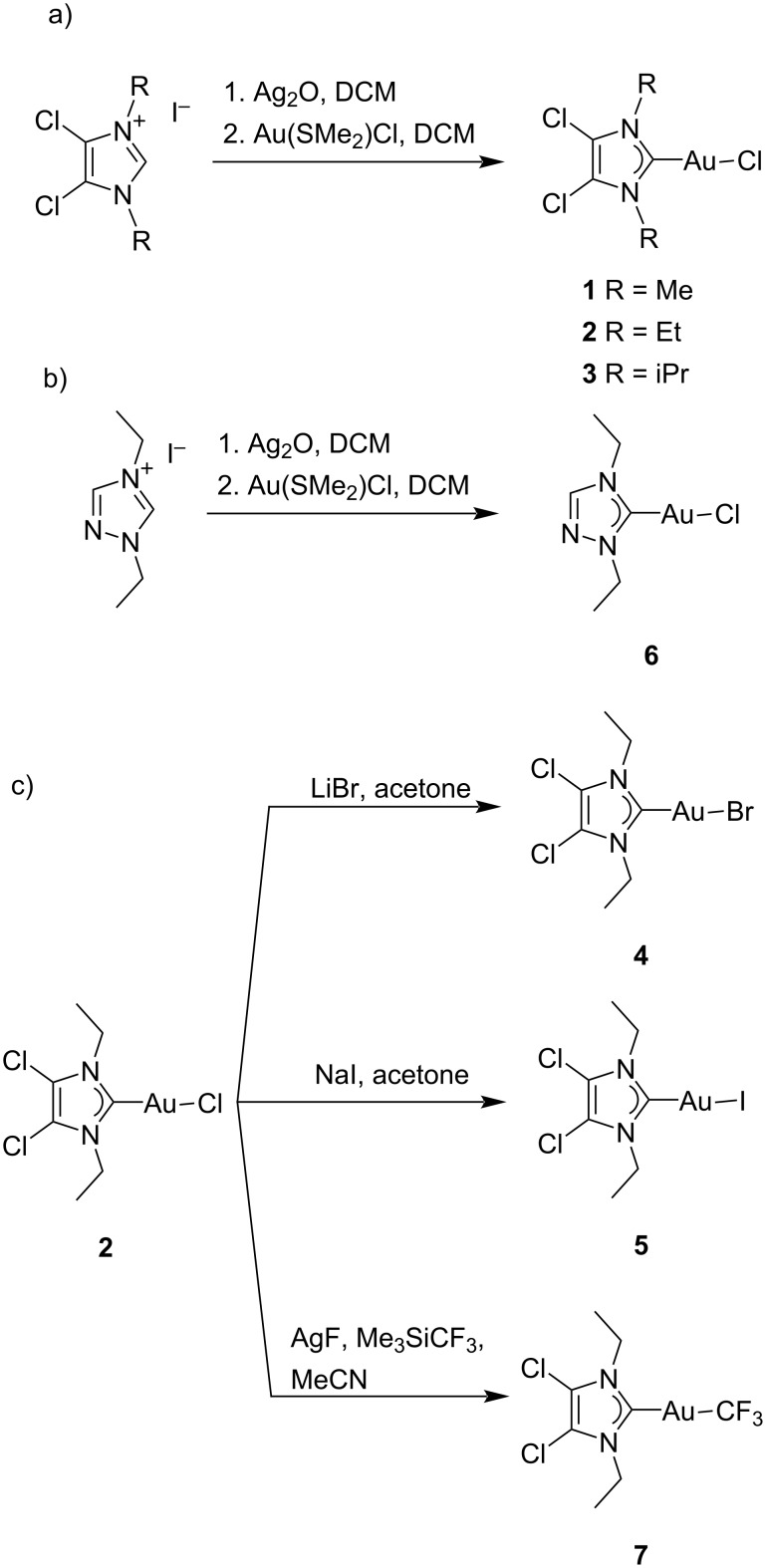
Synthesis routes for **1**–**7**.

The chemical precursors to the selected NHC ligands are salts of their respective imidazolium or triazolium cations. The imidazolium salts were obtained starting from the unsubstituted 4,5-chloroimidazole through two sequential alkylation reactions using the selected alkyl iodides [48]. For the triazolium salt the two alkylation reactions were carried out together in a one-pot synthesis [36]. The resulting salts were then reacted with silver oxide to generate the respective Ag(I) NHC complexes. Upon the addition of 1 equiv of gold precursor Au(SMe_2_)Cl in situ, a transmetalation reaction took place that yielded the desired Au(NHC)Cl complexes **1**, **2**, **3**, and **6** (Scheme 1a,b) [37]. Compounds **4** and **5** were synthesized through a halide metathesis reaction when a solution of **2** in acetone was stirred with a large excess of a bromide [49] or iodide [50] salt (Scheme 1c). Compound **7** was obtained by reaction of **2** with a mixture of AgF and Me_3_SiCF_3_ [51]. This reaction created in situ a AgCF_3_ species that, through a transmetalation reaction, yielded compound **7** (Scheme 1c).

All these compounds are, under ambient conditions, inert colourless crystals or white powders that are stable to air and moisture and safe to handle.

#### Precursor volatility and thermal stability

Table 1 shows the sublimation temperature, obtained from the cold finger setup, for each precursor. No decomposition was observed in the collected sublimed materials by comparison of their ^1^H NMR spectra with those of the bulk material, and no change of the bulk material was observed during the sublimation experiments by visual observation. The correlation between halogen ligand and volatility was recently discussed for a series of isocyanide gold(I) complexes [41]. Halogens with larger radii have been observed to lead to the formation of more volatile complexes, presumably due to the increased intermolecular distances in the packing of the crystals, mainly through weakening of aurophilic interactions [41]. The same trend was observed during cold finger sublimation experiments on the synthesized compounds. However, the magnitude of the sublimation temperature variation was found to be only modest for the halogen series Cl, Br, and I. This can be related to the presence of the NHC moiety, which is predominant in the packing of the molecules, thus reducing the weight of aurophilic interaction in the overall packing energy [52]. The variation of sublimation temperature is more important when a trifluoromethyl group is introduced on the gold atom inducing a decrease of more than 20 °C, in line with observations on isocyanide gold(I) complexes [34]. With the increase of the steric bulk of the N-substituents, the sublimation temperature is decreased. Going from Me to Et substituents causes a decrease in sublimation temperature of 22 °C, while the decrease from Et to iPr is minor (3 °C). Furthermore, the triazole-based gold complex was shown to be more volatile than its imidazole-based counterpart (Table 1).

**Table 1:** Sublimation temperatures, melting points, and the chosen temperature for deposition of the studied precursors.

Compound	Sublimation temperature^a^ (°C)	Melting point^b^ (°C)	Deposition temperature (°C)

(Cl,Me)AuCl (**1**)	100	266–269^c^	120
(Cl,Et)AuCl (**2**)	78	185–186	100
(Cl,iPr)AuCl (**3**)	75	202–203	100
(Cl,Et)AuBr (**4**)	77	204–205	100
(Cl,Et)AuI (**5**)	73	178–179	100
(N,Et)AuCl (**6**)	60	131–132	100
(Cl,Et)AuCF_3_ (**7**)	53	146–149	100

^a^Obtained by cold finger sublimation at 10^−3^ mbar. ^b^Obtained by melting point apparatus. ^c^Decomposition observed starting at 220 °C.

The melting temperatures of the compounds are found not to be correlated directly with the sublimation temperatures. The sublimation temperature is a lower boundary of the FEBID operating range, to achieve a useful vapour pressure. The upper boundary is the upper pressure limit of the SEM. All deposition experiments were performed in this operating range. A temperature of 100 °C was chosen to explore all precursors, except for the least volatile compound **1** for which a temperature of 120 °C (20 °C more than its sublimation temperature) was necessary to provide a sufficient precursor flow. It is noted that all deposits reported in this work are grown on heated substrates and the resulting growth rates are valid only at the corresponding temperature. A beneficial side effect of heating the substrates to at least 100 °C is that adsorbed water is removed from the substrates, diminishing its influence on the FEBID process.

### Testing apparatus

A more flexible setup than a traditional GIS was required for the exploration and testing of a large series of precursors. Commercially available GIS have a limited temperature range. For example, the maximum allowable temperature of the Thermo Fisher Scientific GIS, used with W(CO)_6_ or MeCpPtMe_3_, is limited by the software to 65 °C. As the precursors tested here are inert under normal conditions and stable to air and moisture, they lend themselves for exploration in an open system, and they pose no known health hazard to the user. They were first tested by positioning free precursor crystals on a heated substrate, and observing their disappearance upon heating. Furthermore, electron beam-induced deposition was observed in close vicinity of the crystals. A small and easy to handle setup mounted on a substrate surface was then developed, resembling a GIS, comprising a reservoir, an injection channel, and a deposition area (Figure 2). The cylindrical precursor reservoir of 2.5 mm^3^ (2 mm diameter, 0.8 mm height) and a 1 mm long and 0.5 mm wide channel, which separates the reservoir from the circular deposition area (0.5 mm diameter), were milled from the bottom of an aluminium plate (9.5 × 9.5 × 1 mm^3^). The plate lies directly on the 10 × 10 mm^2^ silicon substrate, covering some precursor crystals positioned on the substrate, and is kept in place with vacuum-compatible Kapton tape. Only small quantities are needed to test a precursor, and a wide range of temperatures can be achieved (tested up to 160 °C).

### Deposition and composition results

**Figure 4:**
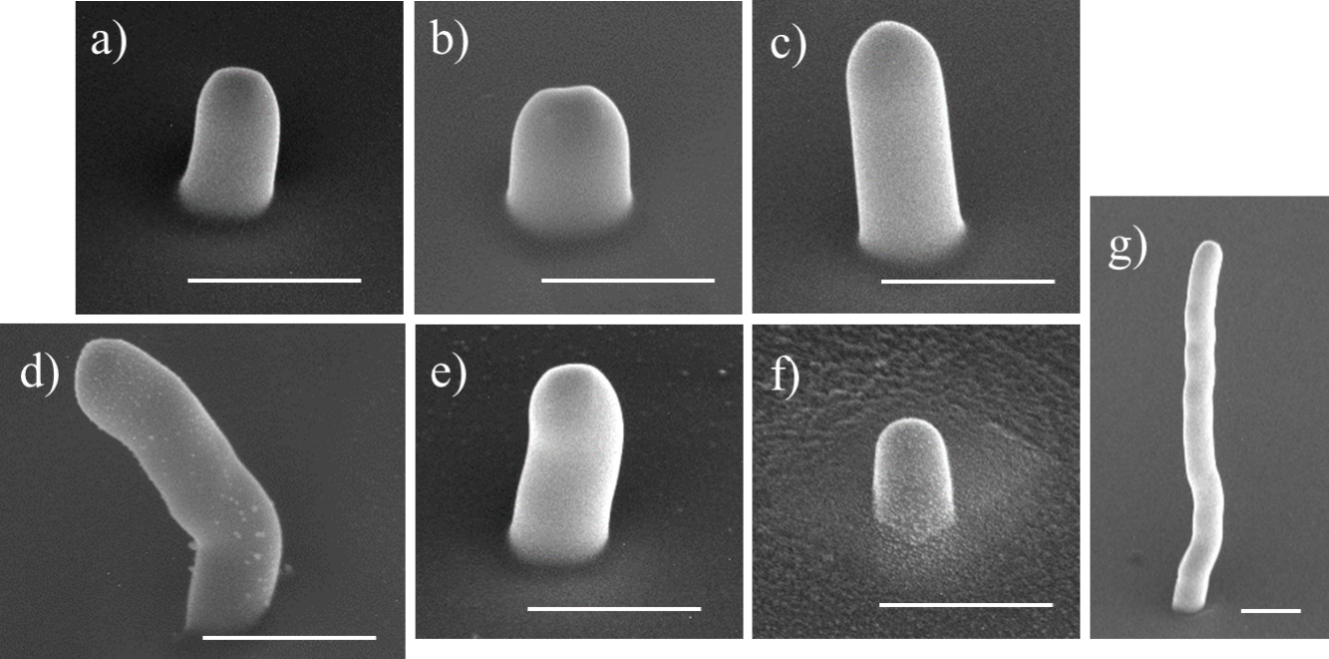
SEM images, tilted by 50°, of 250 × 250 nm^2^ square deposits made using a beam energy and current of 5 keV and 40 pA, respectively. (a) **1** (Cl,Me)AuCl, (b) **2** (Cl,Et)AuCl, (c) **3** (Cl,iPr)AuCl, (d) **4** (Cl,Et)AuBr, (e) **5** (Cl,Et)AuI, (f) **6** (N,Et)AuCl, and (g) **7** (Cl,Et)AuCF_3_. The deposition was performed using the reservoir-on-substrate setup, heated to 100 °C for all compounds except for **1**, which was heated to 120 °C. The scale bars are 1 µm.

All compounds were tested for electron beam-induced deposition, after which deposits were made large enough for compositional analysis using EDX. Slightly larger deposits were grown from the (N,Et)AuCl precursor, in order to reduce the silicon signal from the substrate and to obtain EDX measurements with a silicon content comparable to that of the other studied materials (<10 atom %). Figure 4 shows typical deposits, defined as 250 × 250 nm^2^ squares, from all seven tested precursors. The deposits clearly differ in size and shape. The deposits have grown considerably in the lateral direction, almost doubling in size. Clear differences are seen in the vertical dimension ranging from about 0.5 µm (Figure 4a,f) to 3 µm (Figure 4g). While a minor halo is observed for all precursors, only for (N,Et)AuCl (**6**) a significant granular halo is obtained (see Supporting Information File 1, Figure S19), for reasons yet unknown.

The average composition of seven or eight EDX spectra from at least three deposits as those shown in Figure 4 is listed for each precursor in Table 2. The values reported are the mean ± standard error. All deposits have a consistent atomic fraction (atom %) of gold, indicating the successful delivery of the intact parent molecule to the beam incidence area.

**Table 2:** Elemental composition of deposits obtained by EDX with all values given in atom %. In addition, the ratio between carbon content and gold content in the deposits and the corresponding parent molecules are given.

	(Cl,Me)AuCl **1**	(Cl,Et)AuCl **2**	(Cl,iPr)AuCl **3**	(Cl,Et)AuBr **4**	(Cl,Et)AuI **5**	(N,Et)AuCl **6**	(Cl,Et)AuCF_3_ **7**

C	61.2 ± 0.58	66.7 ± 1.23	68.7 ± 0.96	67.0 ± 0.76	69.0 ± 0.53	60.7 ± 0.61	68.0 ± 0.41
N	14.9 ± 0.38	10.9 ± 0.37	10.0 ± 0.11	11.7 ± 0.30	11.9 ± 0.27	11.3 ± 0.35	12.3 ± 0.32
Au	10.3 ± 0.32	8.8 ± 0.40	7.3 ± 0.15	8.0 ± 0.26	7.3 ± 0.12	14.6 ± 0.41	8.8 ± 0.19
Si	7.4 ± 0.53	6.8 ± 0.67	6.9 ± 0.77	6.8 ± 0.39	7.3 ± 0.50	7.8 ± 0.42	7.2 ± 0.48
O	2.9 ± 0.10	3.7 ± 0.24	4.8 ± 0.25	3.3 ± 0.12	3.3 ± 0.04	4.0 ± 0.16	1.7 ± 0.07
Cl	3.3 ± 0.11	3.1 ± 0.07	2.4 ± 0.08	0.8 ± 0.04	1.3 ± 0.04	1.6 ± 0.08	1.9 ± 0.23
Br	0	0	0	2.5 ± 0.10	0	0	0
F	0	0	0	0	0	0	0.2 ± 0.07
C/Au observed	5.9	7.6	9.4	8.4	9.5	4.2	7.7
C/Au parent^a^	5	7	9	7	7	6	8

^a^C/Au ratio in the parent molecule.

For the series (Cl,Et)AuX with X = Cl, Br, I, and CF_3_ (**2**, **4**, **5**, and **7**), a Au content of 7.3–8.8 atom % was obtained. Although the differences in Au content are only modest, the compounds X = Cl and X = CF_3_ (**2** and **7**) were observed to contain the highest percentage of gold. Going from Cl over Br to I, the Au content steadily decreased. The decrease in metal content from Cl to Br compounds has been observed for deposits from Pt compounds as well [47]. The relatively high chlorine content of compounds **1**–**3**, compared to that of compounds **4**–**7**, indicates that the backbone and the gold-bonded Cl are co-deposited. Br, I, and F are also present in negligible quantities, demonstrating their suitability as elements to be used in FEBID precursors. Iodine is detected in EDX analysis only at 8 keV incident energy, and is therefore not shown in Table 2 (see Supporting Information File 1).

For the series (Cl,R)AuCl with R = Me, Et, and iPr (**1**–**3**) a wider range of gold content was found. This variation can be directly correlated to the number of carbon atoms in the precursor molecule. Going from R = Me over Et to iPr, the Au percentage decreases from 10.3 over 8.8 to 7.3 atom %, respectively, following the trend of the Au/C ratio of the starting material. For all compounds, the C/Au ratio observed in the deposits is comparable to, if not slightly larger than, the ratio in the parent molecules. While the gold composition is highly influenced by the variation of the R substituents, the carbon atomic fraction does not proportionally increase with the increase of carbon atoms in the precursor. Br, Cl, I, and F are partially or mostly removed upon irradiation, while N is mostly co-deposited. A similar behaviour was observed for isocyanide-based gold(I) precursors, where N is also partially co-deposited [34]. Recently, it was further demonstrated that nitrogen can be embedded in pre-existent carbon material upon the use of N-containing precursors under electron irradiation [53]. EDX analyses of the deposits show the presence of silicon and oxygen, which are not present in the precursor molecules. Their presence is likely to come from the silicon substrate with its native oxide surface layer.

The triazole-based compound **6** is the best-performing precursor, yielding the highest Au content of 14.6 atom %. In this case, the C/Au ratio in the deposit is considerably lower than in the parent molecule (Table 2 and Table 3). This could be indicative of the effective fragmentation of the triazole-based ligand. Also in this case, Cl is mostly removed, while N is partially removed, leaving carbon and gold as the mainly deposited atoms.

**Table 3:** Ratios between the atomic percentages of elements present in the precursor molecule of **6**, the deposit from **6** and a putative fragment **6a** (see Figure 5).

	C/Au ratio	N/Au ratio	Cl/Au ratio

parent molecule of **6**	6	3	1
deposit from **6**	4.2	0.8	0.1
fragment **6a**	4	1	0

**Figure 5:**
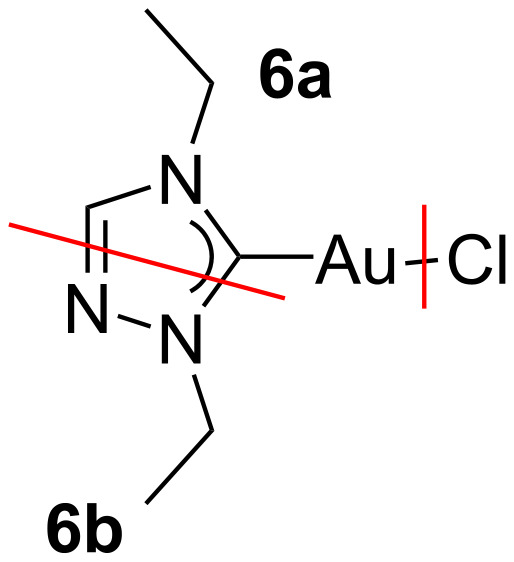
Possible fragmentations of (N,Et)AuCl (**6**), with the loss of Cl and a volatile fragment **6b,** and the deposition of **6a**.

Based on the N/Au, C/Au, and Cl/Au ratios for compound **6** (Table 3), it is evident that the chloride ligand is removed and that the triazole-ring is fragmented. However, the fragmentation of the NHC ligand is not simple and unambiguous, as various fragmentations can take place either by the removal of the N-substituents or by the effective fragmentation of the NHC ring. Since no fragmentation of the ring occurs for the imidazole-based compounds, the presence of a N–N bond appears to be a requirement. Thus, we postulate that the most plausible fragmentation is the removal of a N=N–Et fragment (**6b**) with the co-deposition of the metal centre and the strongly bonded carbene moiety **6a**, for which the molecular structure is comparable to the imidazole-based precursors (Figure 5). However, we cannot rule out the possibility of different fragmentations occurring at the same time.

### Growth Rate

**Figure 6:**
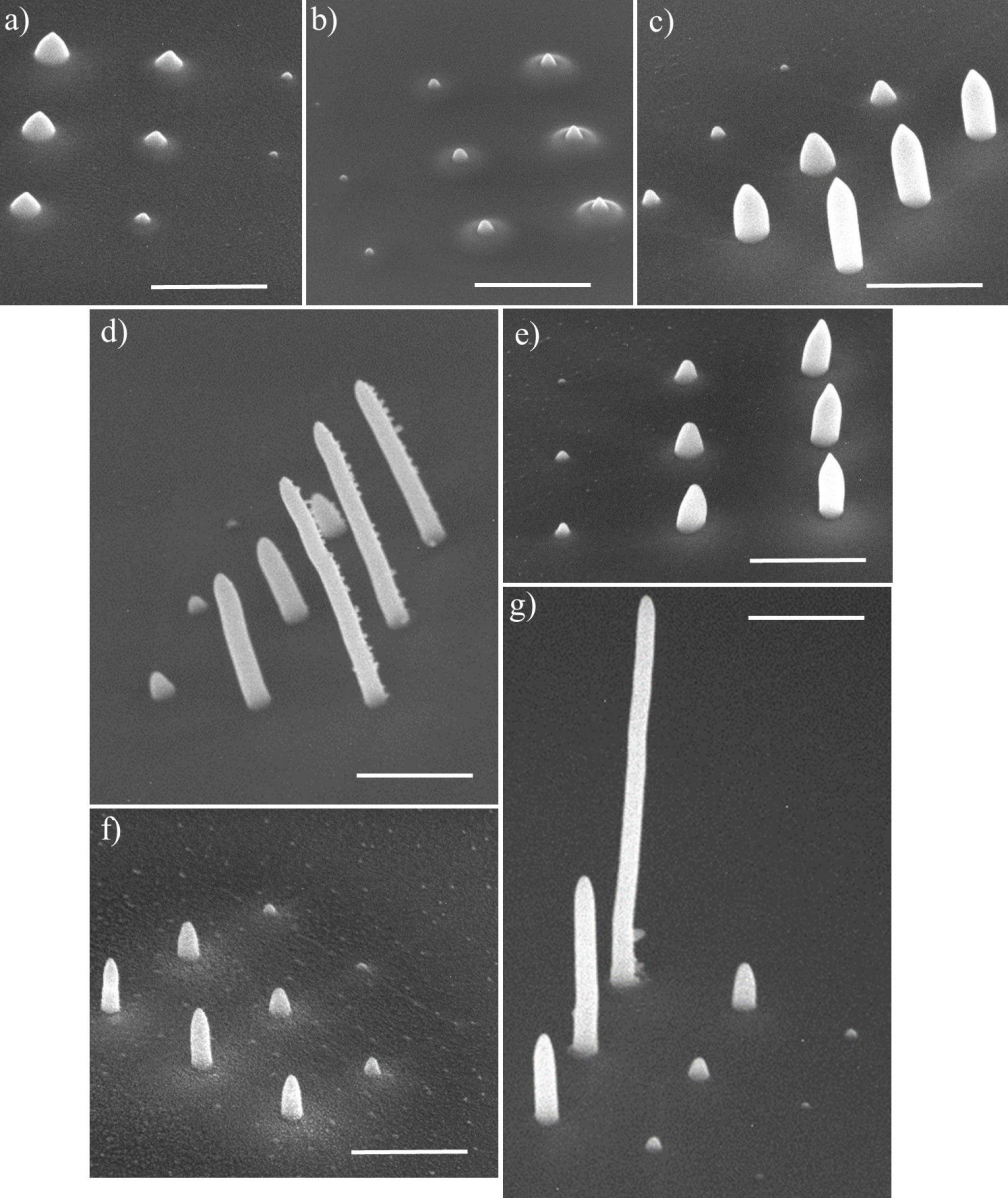
Typical SEM images, tilted by 50°, of pillar arrays deposited from all precursors. (a) **1** (Cl,Me)AuCl, (b) **2** (Cl,Et)AuCl, (c) **3** (Cl,iPr)AuCl, (d) **4** (Cl,Et)AuBr, (e) **5** (Cl,Et)AuI, (f) **6** (N,Et)AuCl, and (g) **7** (Cl,Et)AuCF_3_. The deposition was performed using the reservoir-on-substrate setup heated to 100 °C for all compounds except for **1**, which was heated to 120 °C. The scale bars are 1 µm.

To evaluate the deposition rate of these novel precursors, square arrays of 3 × 3 pillars were deposited, with each pillar grown with a different deposition time as explained in the Experimental section. Arrays with short deposition times (type 1) were deposited from (Cl,Et)AuCF_3_, due to its high deposition rate. For all other precursors, arrays with long deposition times (type 2) were fabricated. Figure 6 shows SEM tilt images (at 5 keV and 40 pA) of a typical array of deposited pillars for each precursor. For each deposited pillar its total deposition time is converted to the total number of incident electrons, or electron dose, used to deposit that pillar.

**Figure 7:**
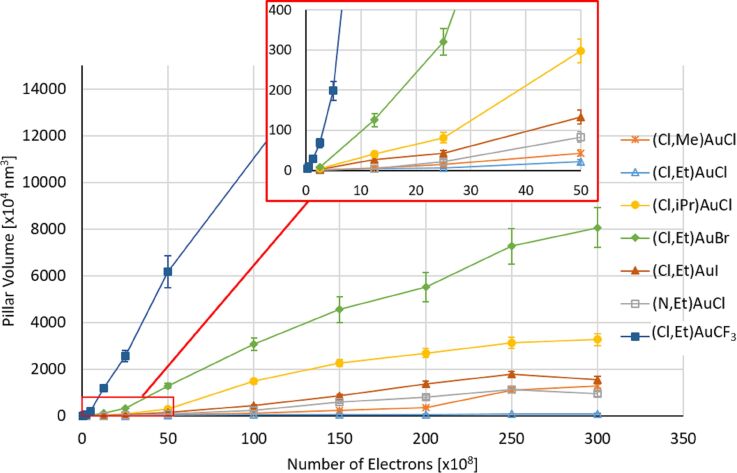
Calculated volume of pillars grown using a 5 kV, 40 pA beam, as a function of the electron dose given as total number of primary electrons used to deposit a pillar. During all experiments the substrate and the precursor were heated together to 100 °C, except for (Cl,Me)AuCl, which was heated to 120 °C. For each precursor an array of 3 × 3 pillars was deposited. The lines between the points merely serve as a guide to the eye.

Figure 6 shows that the shapes of the pillar deposits from different precursors are different. Therefore, the volume was chosen to fairly compare the growth rate of the different precursors. The height and diameter of the pillars were measured and used to calculate the volume. Figure 7 shows the calculated deposit volumes as a function of the electron dose. Clearly different deposition rates are found ranging from 3 × 10^−5^ to 1 × 10^−2^ nm^3^/e^−^, assuming linear growth. For the well-known Pt precursor MeCpPtMe_3_, the deposition rate was recently reported as 2 × 10^−2^ nm^3^/e^−^, although deposited under very different conditions [47]. Except for the compound (Cl,Et)AuCF_3_, the growth rates of the presently studied gold precursors are considerably lower, which may be due to the elevated substrate temperature at which the deposition was performed. Arranging the precursors according to their growth rate, in increasing order, the following sequence is obtained: (Cl,Et)AuCl < (Cl,Me)AuCl < (N,Et)AuCl < (Cl,Et)AuI < (Cl,iPr)AuCl < (Cl,Et)AuBr < (Cl,Et)AuCF_3_. No clear correlation between sublimation temperature and growth rate is observed. However, it should be noted that the most volatile compound (Cl,Et)AuCF_3_ (**7**) led to the highest growth rate. Height and diameter of pillars grown from all precursors **1**–**7**, used to calculate the volume, are plotted in Figure S20 in Supporting Information File 1. Deposits from all precursors show a rather linear increase in height with electron dose (Figure S20a, Supporting Information File 1), whereas the deposit diameter increases at first but saturates at higher doses (Figure S20b, Supporting Information File 1). The saturation values differ between precursors, probably reflecting the difference in composition, which influences the electron scattering in the pillars.

## Conclusion

Seven gold NHC complexes of the form (Y,R)AuX were synthesized and thermally characterized. The sublimation temperature *T*_s_ of the compounds was observed to decrease with the increase of the steric bulk of the N-substituent in the series R = Me, Et, and iPr, that is, *T*_s,Me_ > *T*_s,Et_ > *T*_s,iPr_, and with the variation of the halide ligand X in the series X = Cl , Br, I, and CF_3_, that is, *T*_s,Cl_ ≈ *T*_s,Br_ > *T*_s,I_ > *T*_s,CF3_. Minor structural variations were observed to cause a sublimation temperature difference of 47 °C between the least volatile compound (Cl,Me)AuCl (**1**) and the most volatile compound (Cl,Et)AuCF_3_ (**7**). Furthermore, the introduction of a triazole-based ring in (N,Et)AuCl (**6**) leads to a more volatile complex than the imidazole-based counterpart (Cl,Et)AuCl (**2**). The compounds were tested as FEBID precursors on a heated substrate equipped with an on-substrate precursor reservoir. We analyzed the influence of the variation of the NHC ring, of the N-substituents and of the halogen or pseudo halogen ligand X both on composition and deposition rate. The variation of the R group expectedly led to better composition results for the smaller alkyl substituents (R = Me), accompanied by a decrease in volatility. Minor composition differences were registered for the variation of the X group, with X = Cl and CF_3_ leading to the best results. Of the tested precursors, the most promising is the triazole-based complex **6**, which leads to an un-optimized gold composition of 14.6 atom %. Modest atomic percentages of gold have been achieved, in line with previously reported gold(I) precursors, but still below the best-performing unstable gold(I) precursors and Au(tfac)Me_2_. However, there is much room for improvement regarding the deposition conditions, which could lead to better composition of the deposits. The growth rate measurements of the compounds, at the temperature of deposition, have shown that under constant conditions the variation of the X group from a halogen to a trifluoromethyl group is highly beneficial. (Cl,Et)AuCF_3_ (**7**) shows the highest growth rate while retaining the same gold composition as the halogen-based complexes. This comparison offers an interesting perspective to further explore trifluoromethylated FEBID precursors, rather than the halide counterparts.

## Supporting Information

Supporting Information contains contains NMR characterization of **4**–**7**, ^1^H NMR spectra of sublimation experiments of **1**–**7**, and a graphical representation of vertical growth and diameter of the pillars in Figure 6.

File 1 Additional experimental data.
