# Supplementary material for: Gold(I) N-heterocyclic carbene precursors for focused electron beam-induced deposition
Source: Beilstein J Nanotechnol. 2021 Mar 17;12:257–69. doi: 10.3762/bjnano.12.21 (PMC7991619; doi:10.3762/bjnano.12.21)
Supplement: File 1 — Additional experimental data. [file Beilstein_J_Nanotechnol-12-257-s001.pdf]

BEILSTEIN JOURNAL OF NANOTECHNOLOGY

Supporting Information

for

Gold(I) N-heterocyclic carbene precursors for focused electron beam-induced deposition

Cristiano Glessi, Aya Mahgoub, Cornelis W. Hagen and Mats Tilset

Beilstein J. Nanotechnol. **2021,** *12,* 257–269. doi:10.3762/bjnano.12.21

Additional experimental data

License and Terms: This is a supporting information file under the terms of the Creative Commons Attribution License (https://creativecommons.org/licenses/by/4.0). Please note that the reuse, redistribution and reproduction in particular requires that the author(s) and source are credited and that individual graphics may be subject to special legal provisions.
The license is subject to the *Beilstein Journal of Nanotechnology* terms and conditions: (https://www.beilstein-journals.org/bjnano/terms)

Table of content:

NMR characterization and cold finger sublimation experiments of the precursors **1**–**7** S3

1 (Cl,Me)AuCl S3

2 (Cl,Et)AuCl S4

3 (Cl,iPr)AuCl S5

4 (Cl,Et)AuBr S6

5 (Cl,Et)AuI S9

6 (N,Et)AuCl S12

7 (Cl,Et)AuCF_3 ....S16

Supplemental deposition data for **1**–**7** S19

NMR characterization and cold finger sublimation experiments of the precursors 1–7

1 (Cl,Me)AuCl

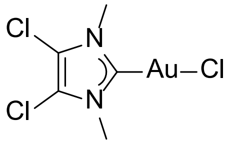

Sublimation. From top to bottom in order: **1**, sublimed obtained for three different experiments at a registered temperature of: 100.7 °C, 99.6 °C, 100.9 °C.

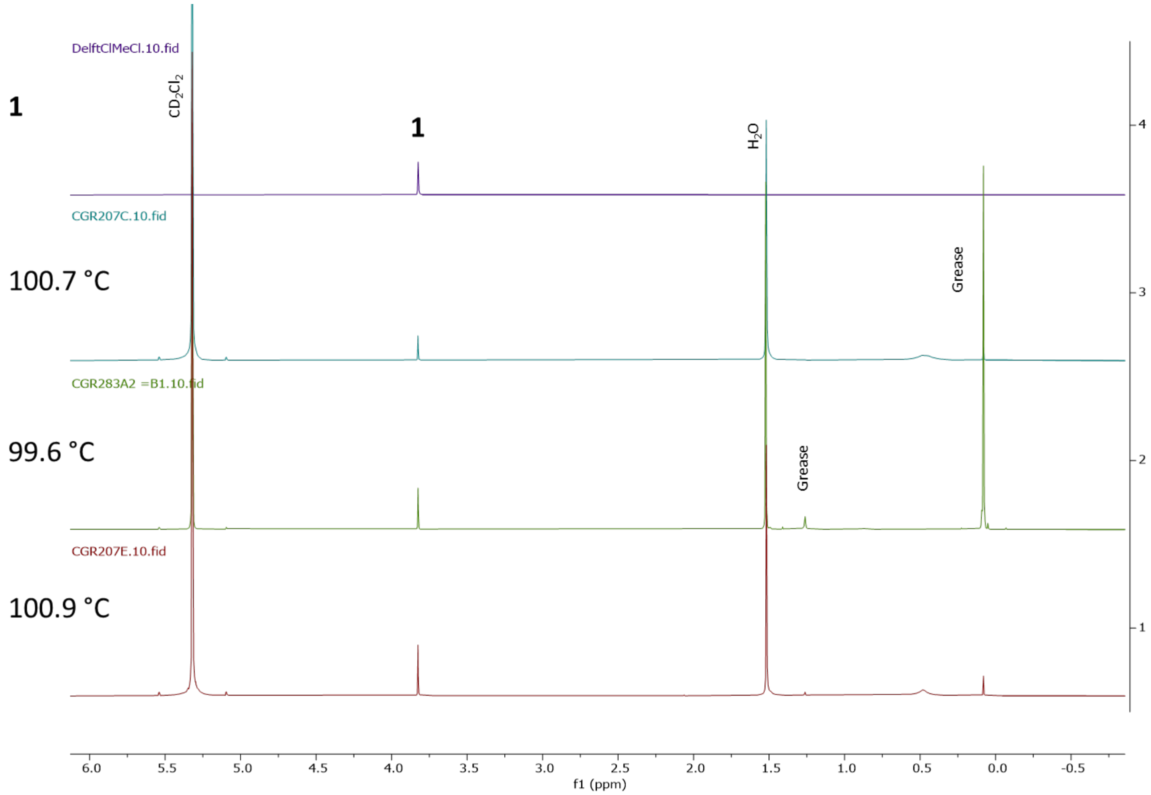

Figure S1: ^{1}H NMR spectra stack plot for **1** and the obtained sublimation materials (400 MHz or 300 MHz, CD_2Cl_2).

2 (Cl,Et)AuCl

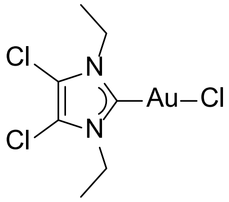

Elemental analysis: Calcd. for $C_7H_{10}AuCl_3N_2$: C, 19.76; H, 2.37; N, 6.58%. Found: C, 19.80; H, 2.35; N, 6.56%.

Sublimation. From top to bottom in order: **2**, sublimed obtained for three different experiments at a registered temperature of: 78.3 °C, 78.3 °C, 78.2 °C.

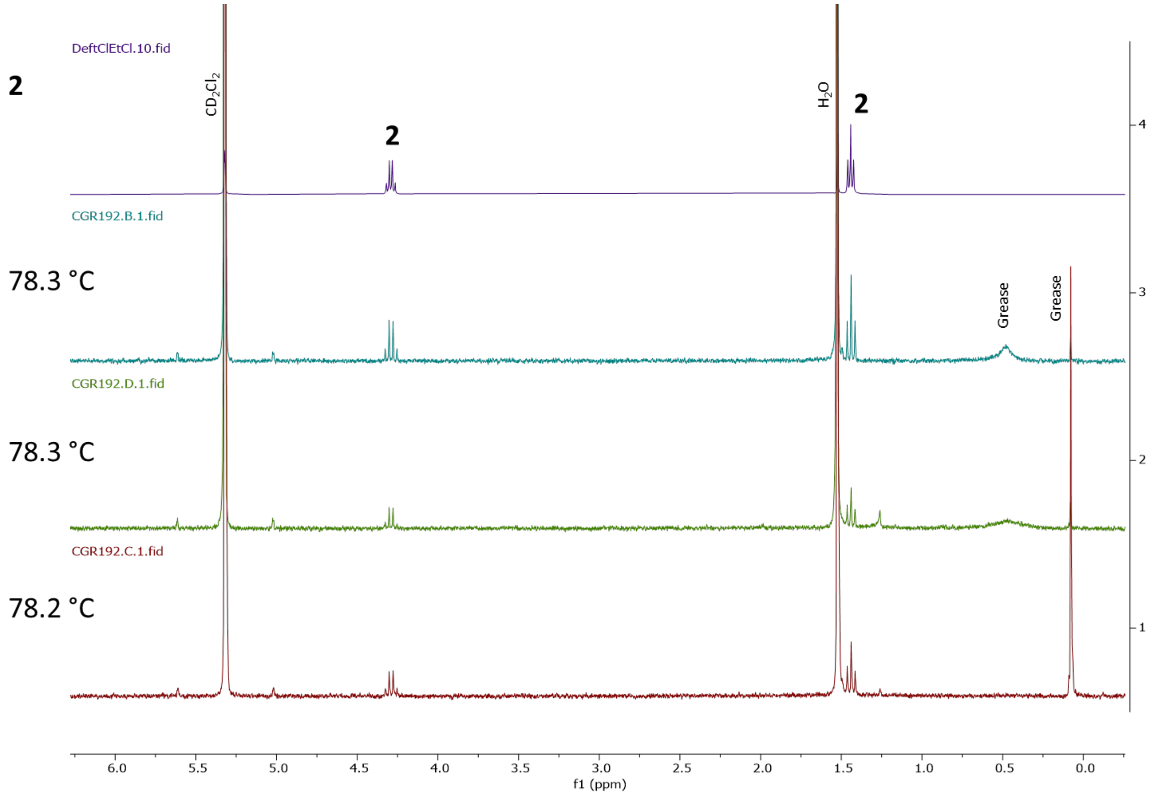

Figure S2: ^{1}H NMR spectra stack plot for **2** and the obtained sublimation materials (400 MHz or 300 MHz, CD_2Cl_2).

3 (Cl,iPr)AuCl

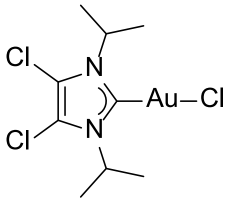

Elemental analysis: Calcd. for $C_9H_{14}AuCl_3N_2$: C, 23.83; H, 3.11; N, 6.18%. Found: C, 23.81; H, 3.09; N, 6.19%.

Sublimation. From top to bottom in order: **3**, sublimed obtained for three different experiments at a registered temperature of: 75.2 °C, 76.2 °C, 75.2 °C.

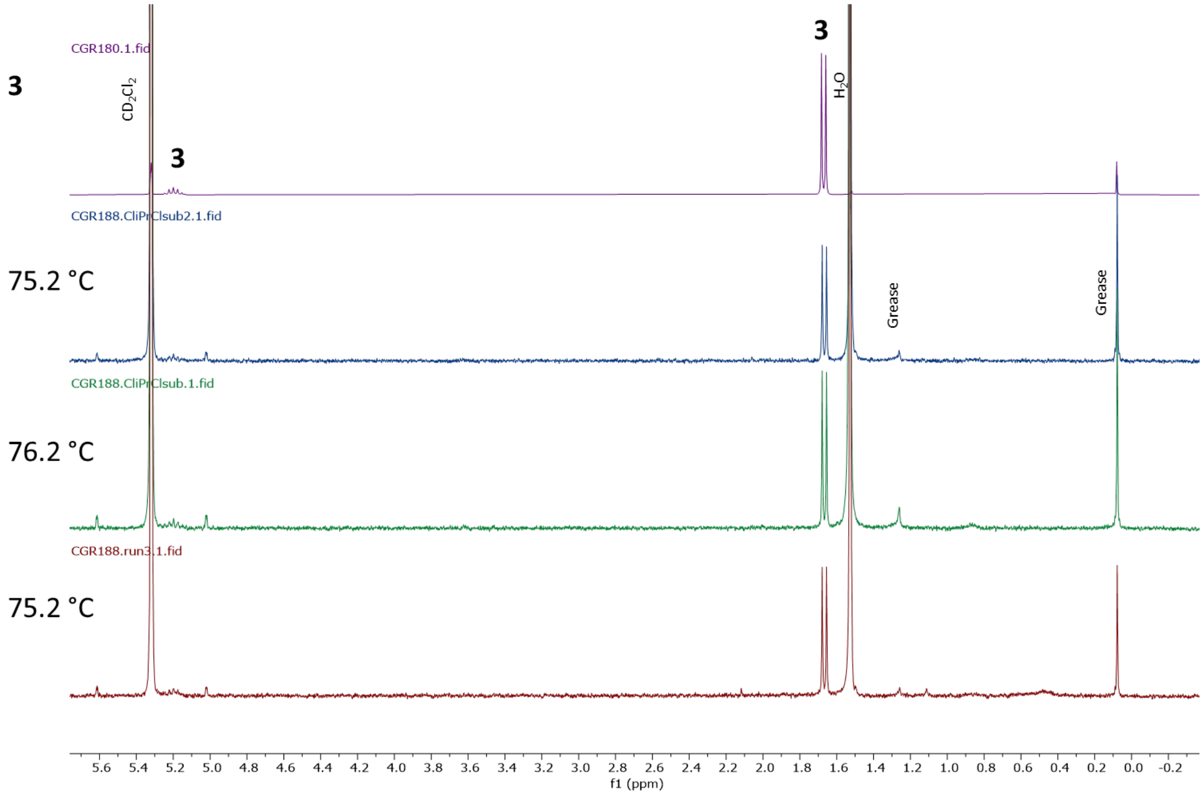

Figure S3: ^1H NMR spectra stack plot for **3** and the obtained sublimation materials (400 MHz or 300 MHz, CD_2Cl_2).

4 (Cl,Et)AuBr

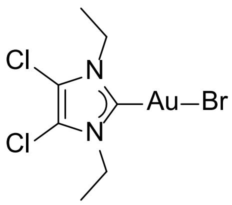

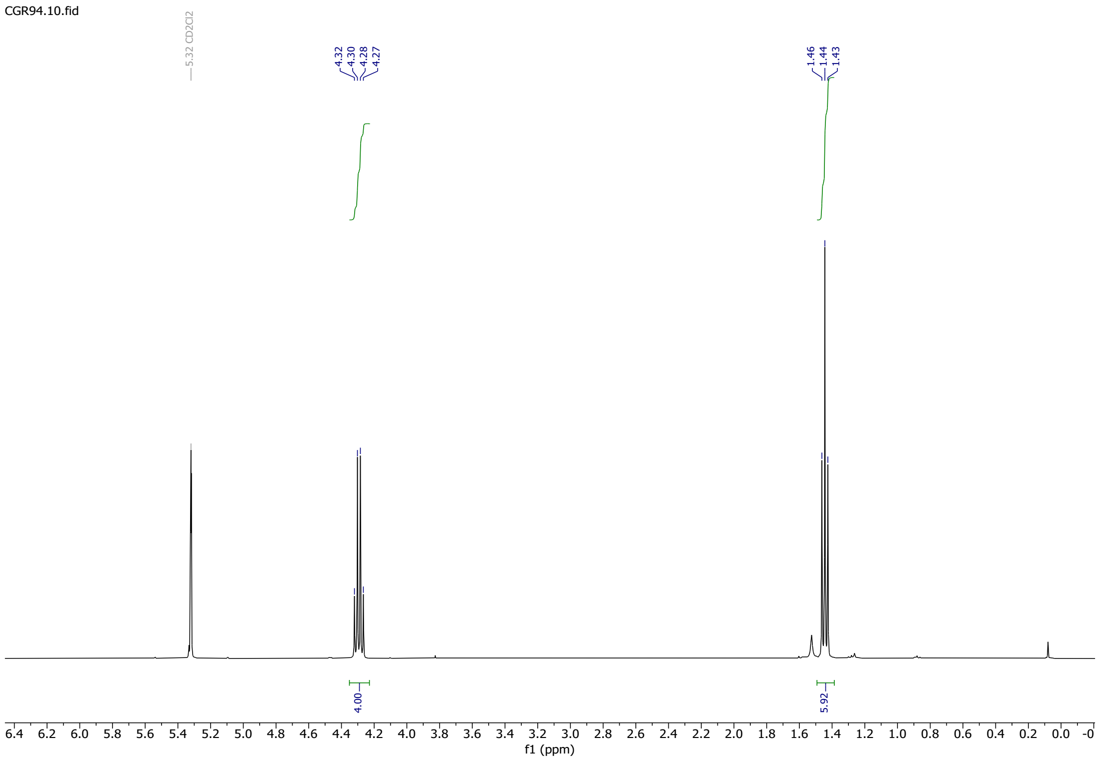

Figure S4: ^{1}H NMR spectrum of **4** (400 MHz, CD_2Cl_2).

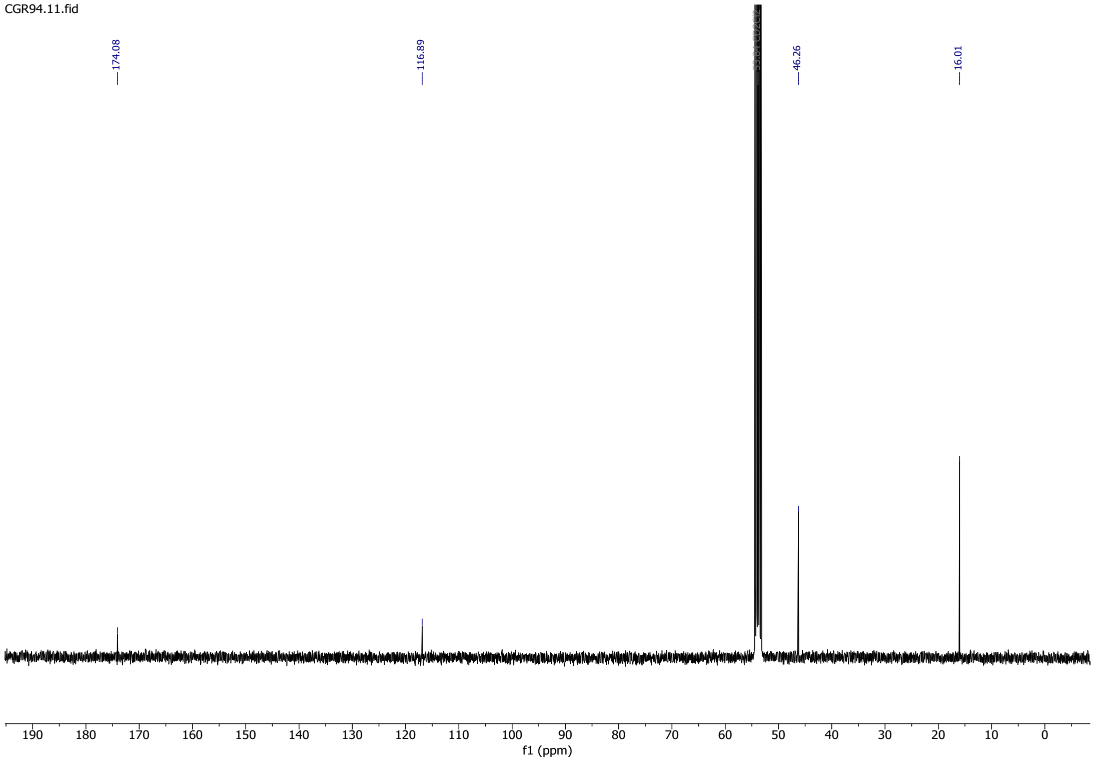

Figure S5: ^{13}C NMR spectrum of **4** (101 MHz, CD_2Cl_2).

Sublimation. From top to bottom in order: **4**, sublimed obtained for three different experiments at a registered temperature of: 77.3 °C, 77.2 °C, 77.4 °C.

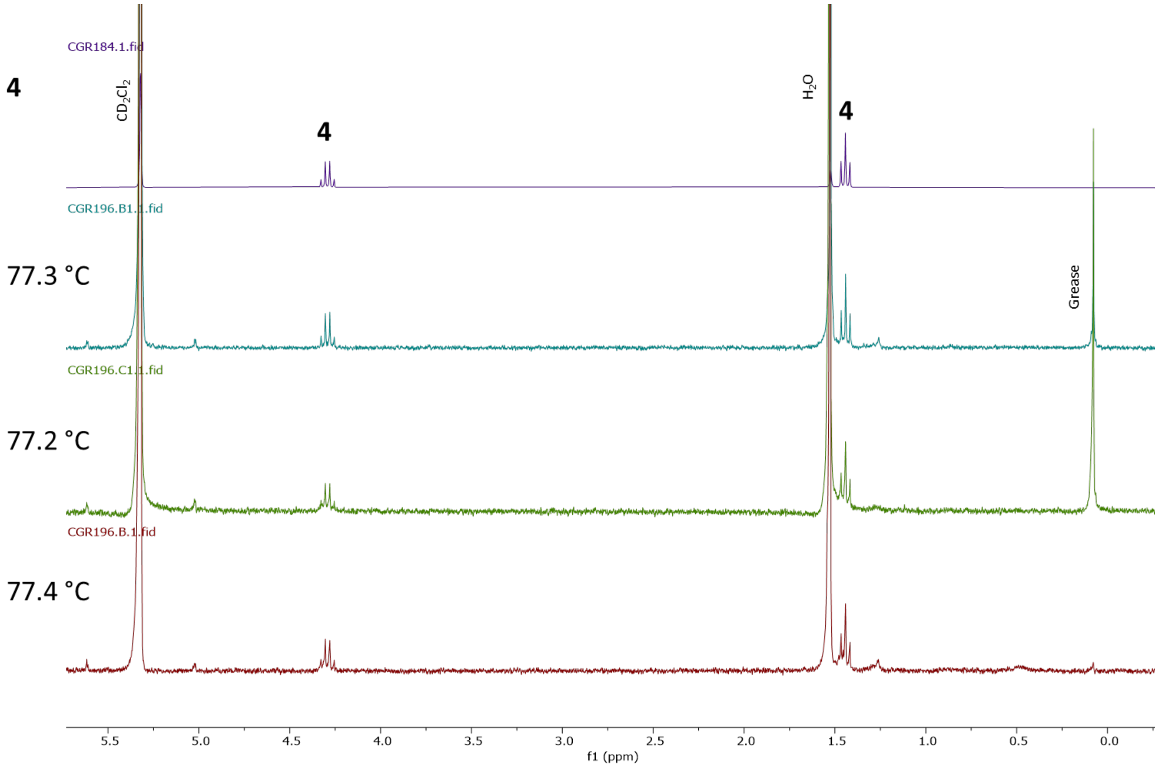

Figure S6: ^{1}H NMR spectra stack plot for **4** and the obtained sublimation materials (400 MHz or 300 MHz, CD_2Cl_2).

5 (Cl,Et)AuI

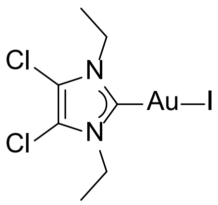

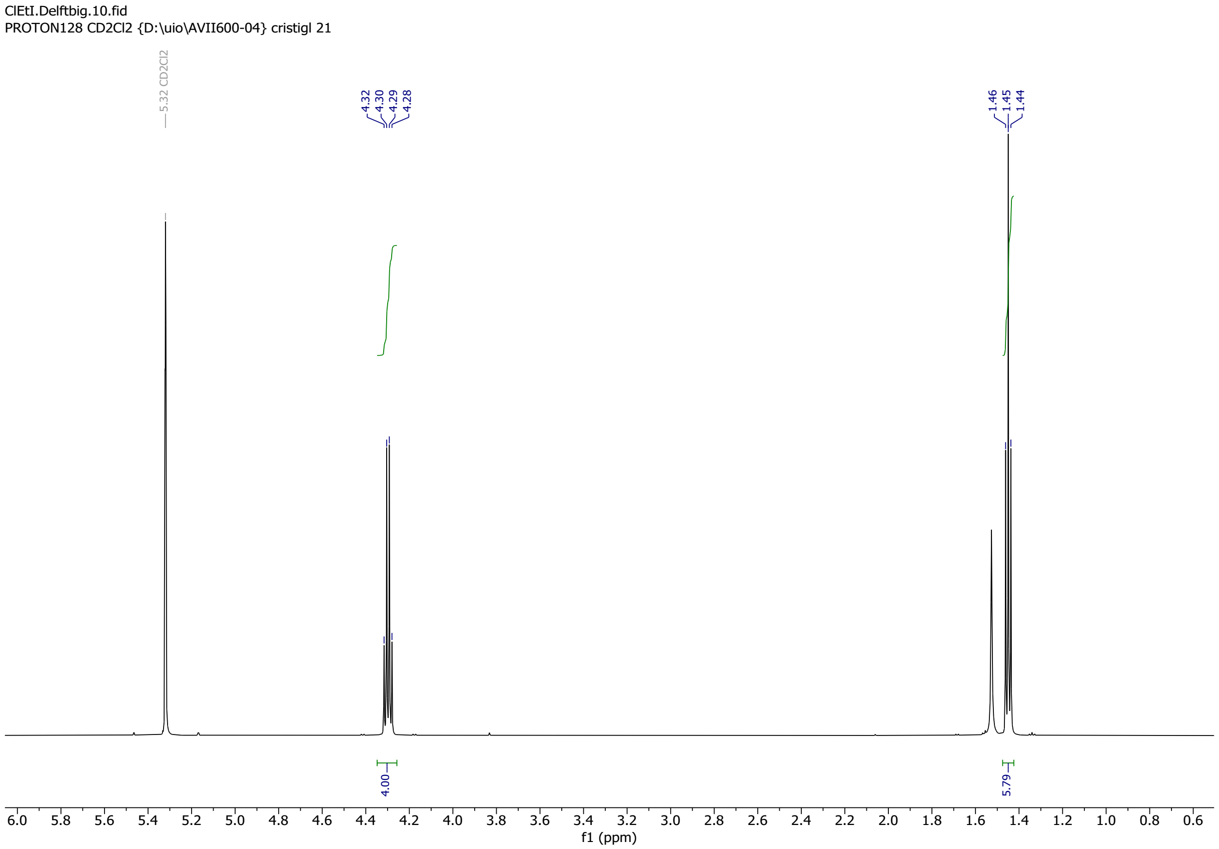

Figure S7: ^{1}H NMR spectrum of **5** (600 MHz, CD_2Cl_2).

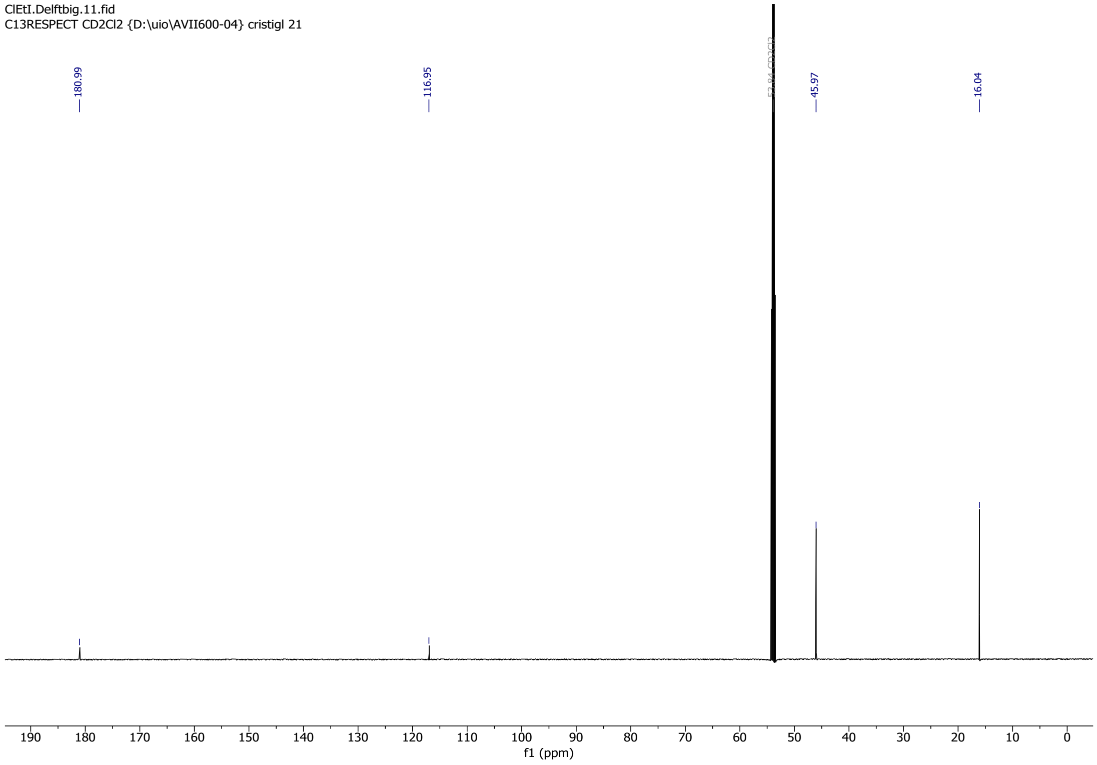

Figure S8: ^{13}C NMR spectrum of **4** (151 MHz, CD_2Cl_2).

Sublimation. From top to bottom in order: **5**, sublimed obtained for three different experiments at a registered temperature of: 73.3 °C, 73.2 °C, 73.3 °C.

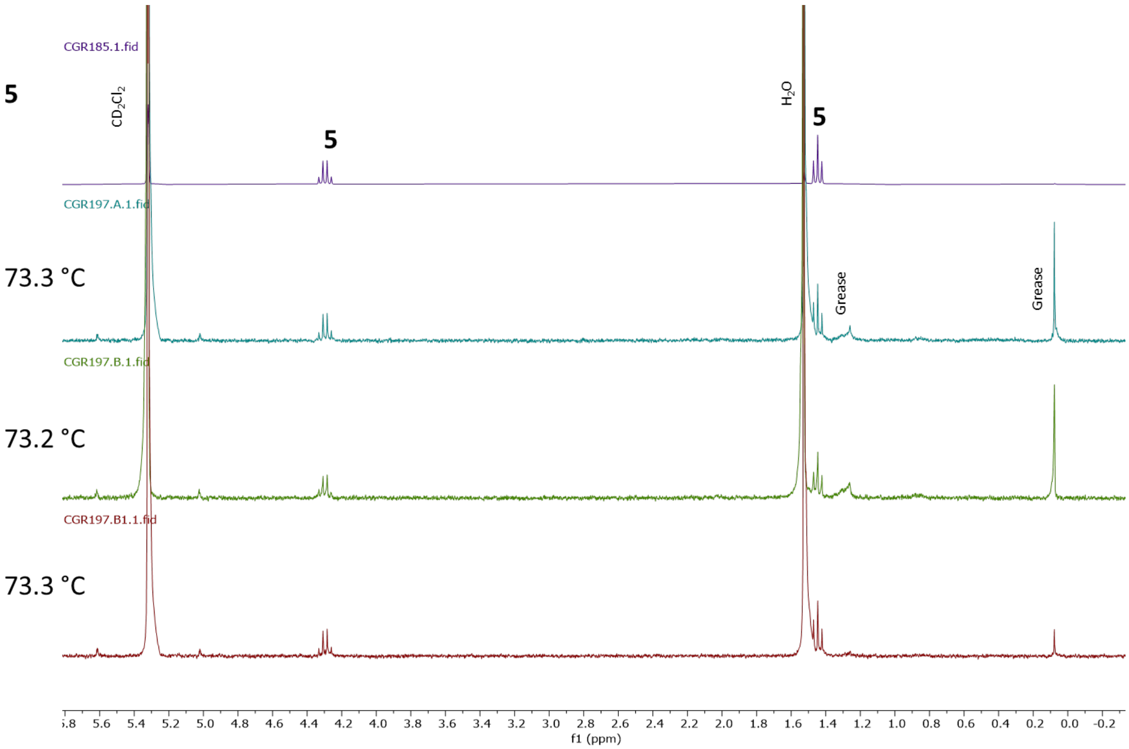

Figure S9: ^{1}H NMR spectra stack plot for **5** and the obtained sublimation materials (400 MHz or 300 MHz, CD_2Cl_2).

6 (N,Et)AuCl

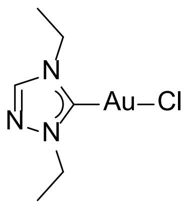

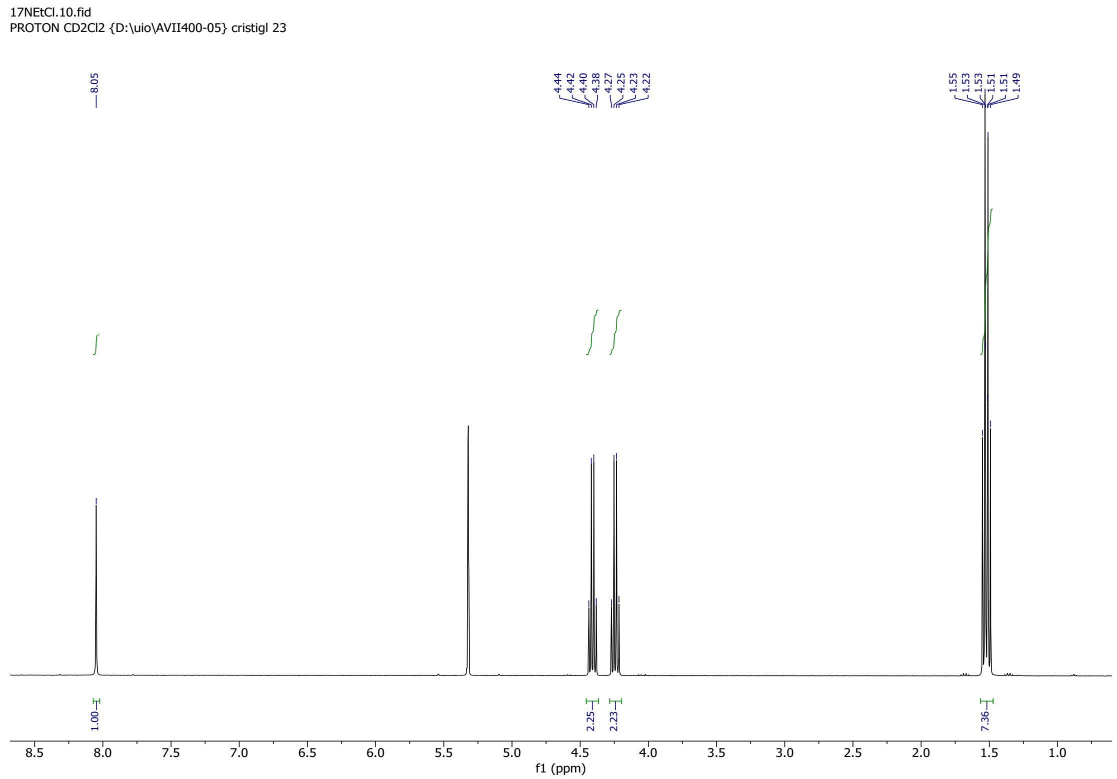

Figure S10: ^{1}H NMR spectrum of 6 (400 MHz, CD_2Cl_2).

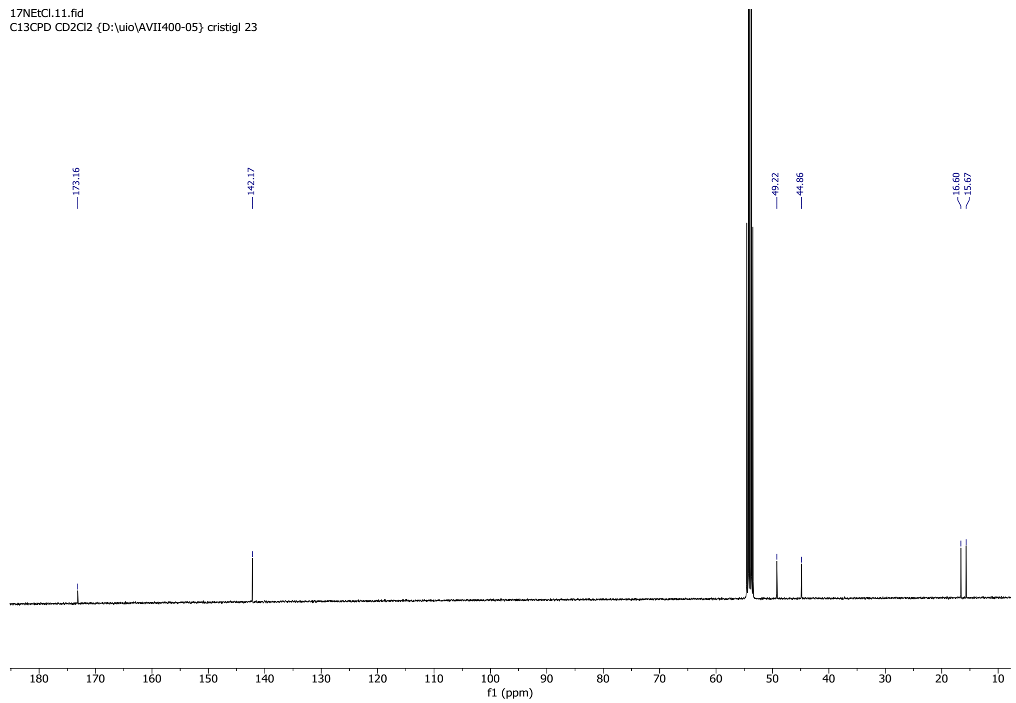

Figure S11: ^{1}H NMR spectrum of **6** (101 MHz, CD_2Cl_2).

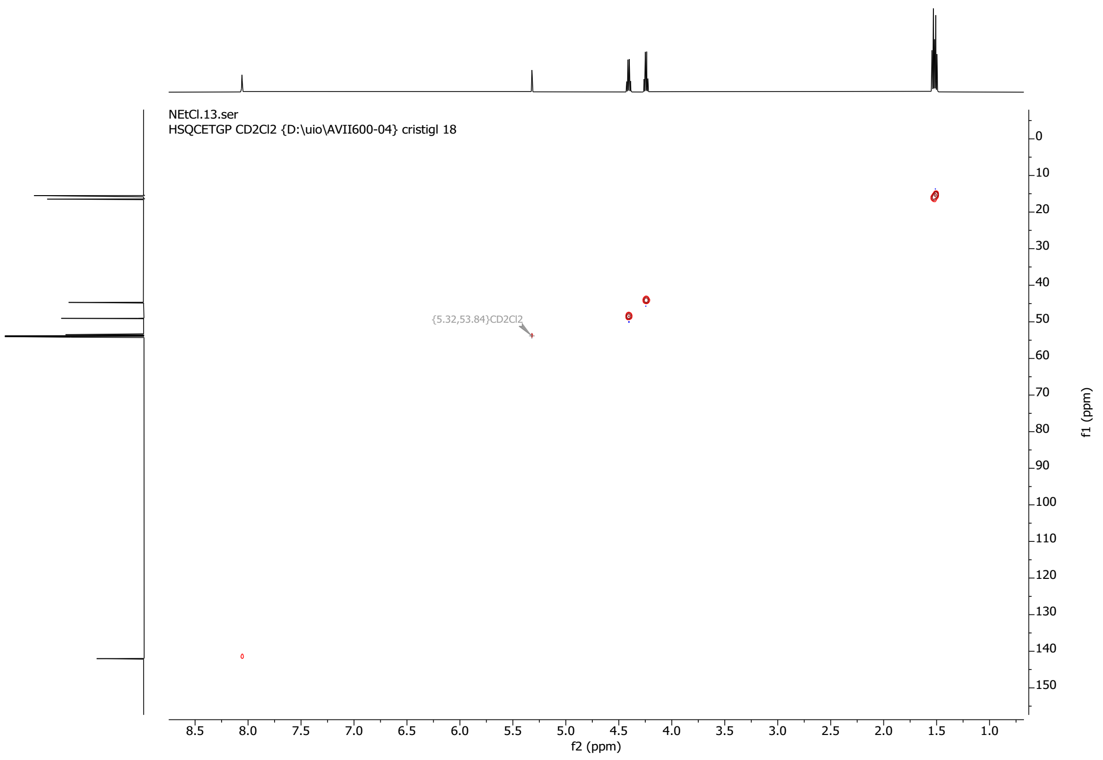

Figure S12: ^1H–^{13}C HSQC spectrum of **6** (600 MHz, CD_2Cl_2).

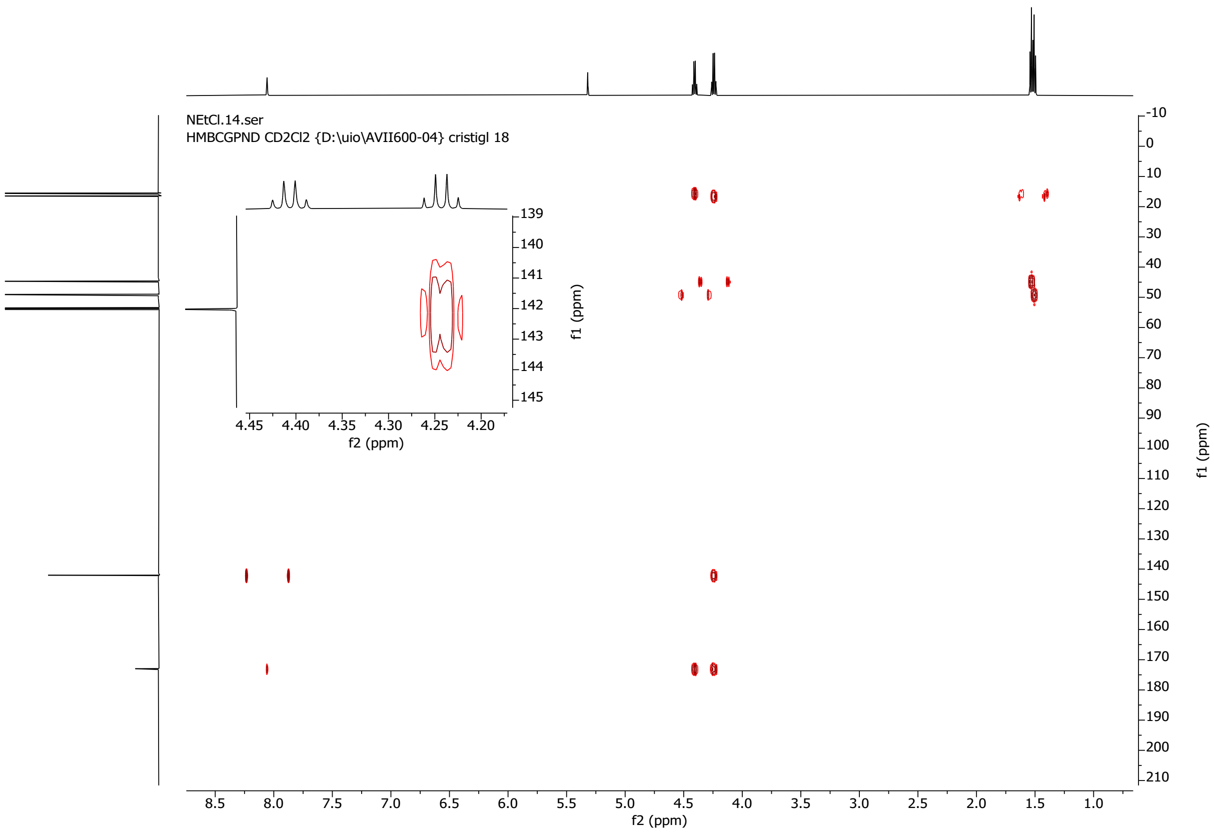

Figure S13: ^{1}H–^{13}C HMBC spectrum of **6** (600 MHz, CD_2Cl_2).

Sublimation. From top to bottom in order: **6**, sublimed obtained for three different experiments at a registered temperature of: 60.2 °C, 59 °C, 60.2 °C.

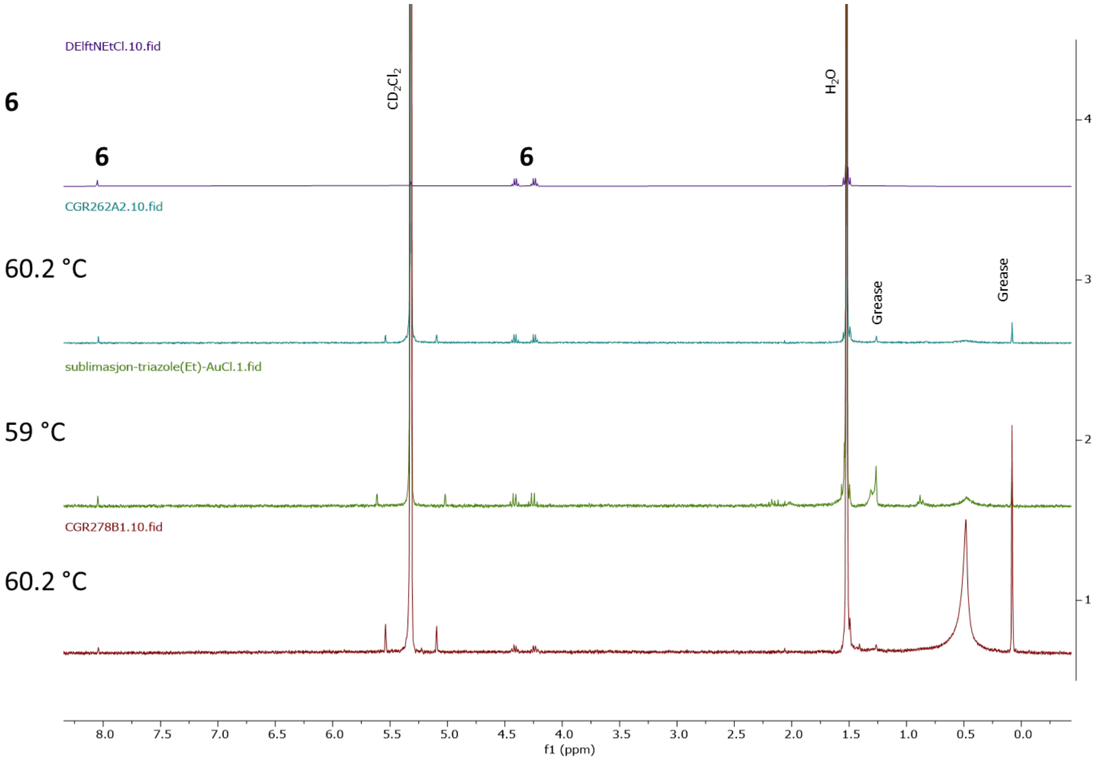

Figure S14: ^{1}H NMR spectra stack plot for **6** and the obtained sublimation materials (400 MHz or 300 MHz, CD_2Cl_2).

7 (Cl,Et)$AuCF_3$

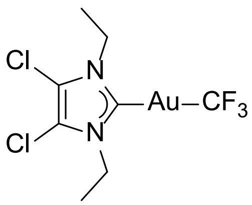

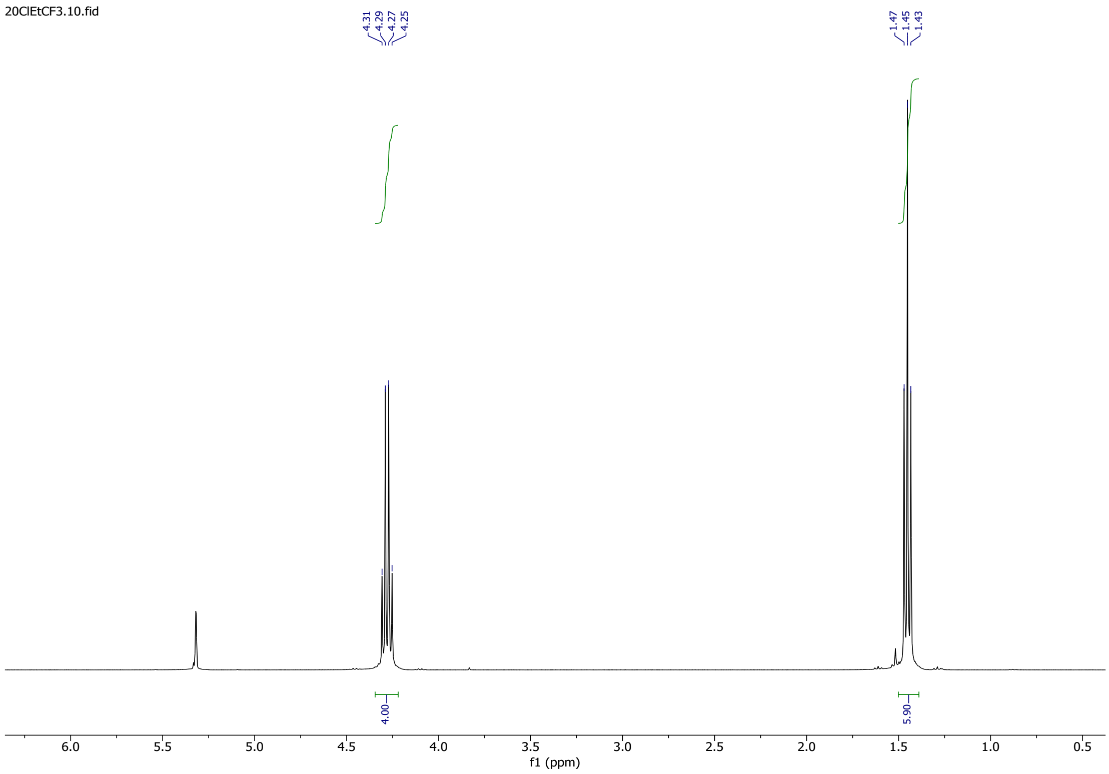

Figure S15: ^1H NMR spectrum of **6** (400 MHz, CD_2Cl_2).

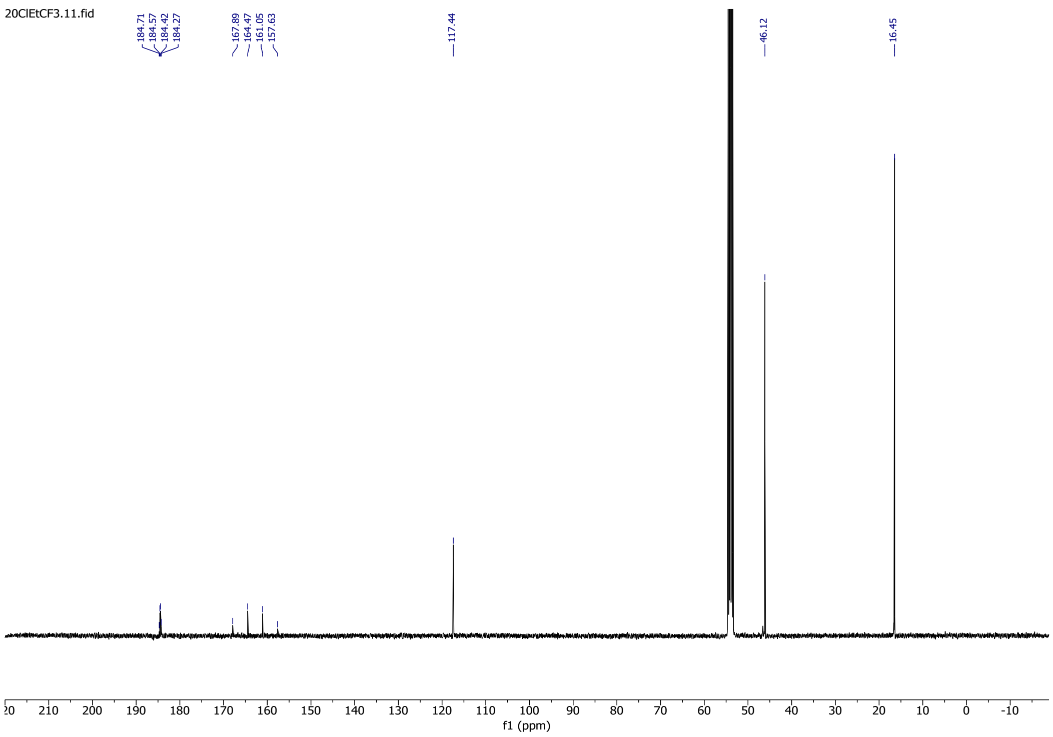

Figure S16: ^{13}C NMR spectrum of **6** (101 MHz, CD_2Cl_2).

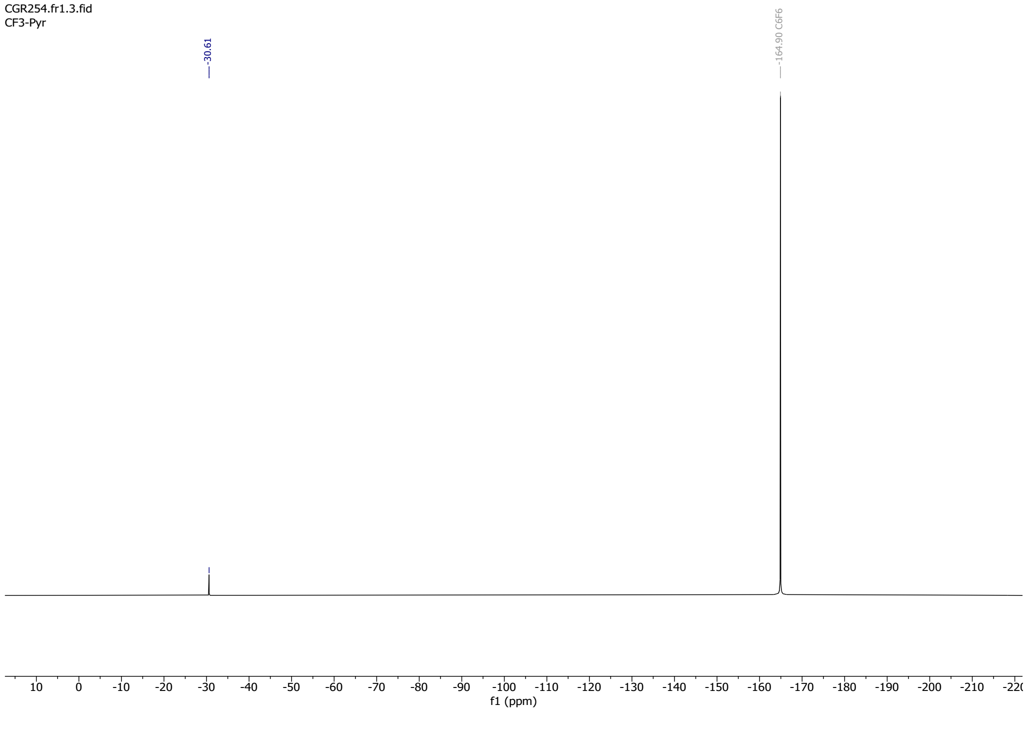

Figure S17: ^{19}F NMR spectrum of **6** (188 MHz, CD_2Cl_2).

Sublimation. From top to bottom in order: **6**, sublimed obtained for three different experiments at a registered temperature of: 54.9 °C, 52.8 °C, 53.1 °C. For the first experiment no heating rate is applied as immediate sublimation is observed.

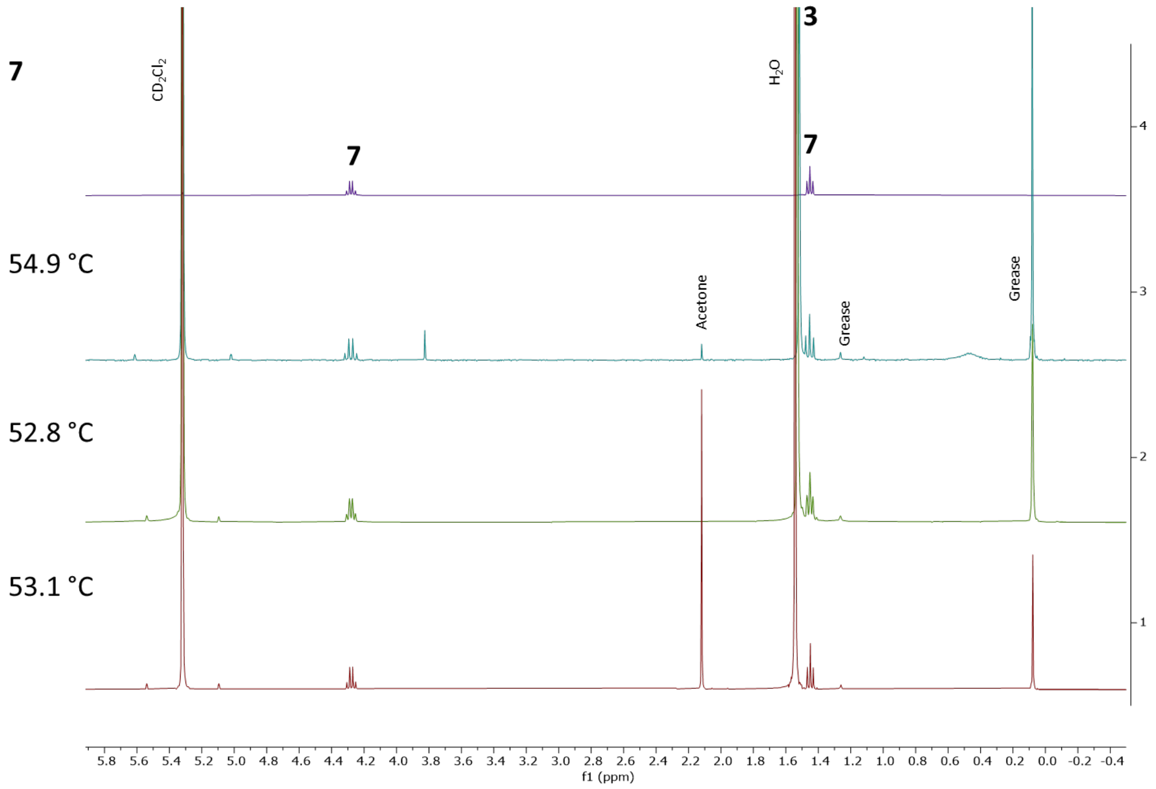

Figure S18: ^{1}H NMR spectra stack plot for **7** and the obtained sublimation materials (400 MHz or 300 MHz, CD_2Cl_2).

Supplemental deposition data for 1–7

Table S1: EDX of 5 performed at 8 keV and 600 pA.

Element	Average	Standard error
C	65.4	0.23
N	10.5	0.23
Au	5.0	0.07
Si	13.4	0.13
O	2.8	0.04
Cl	0.9	0.02
I	2.0	0.05

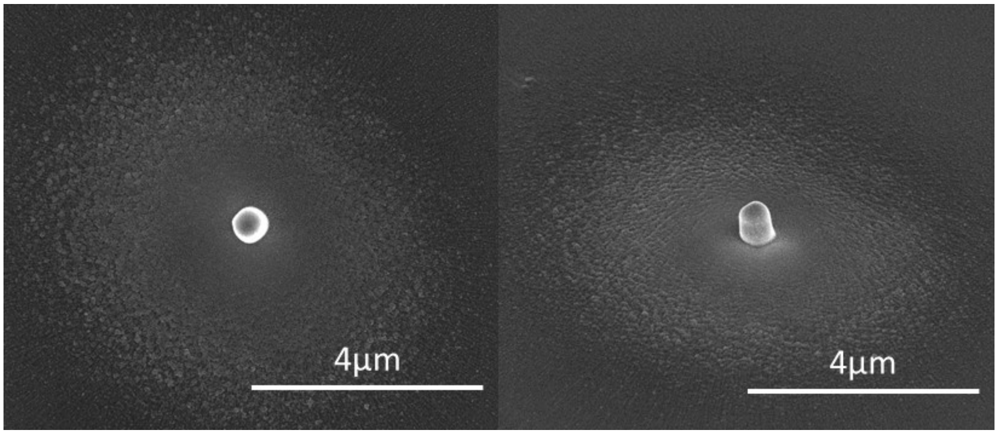

Figure S19: 250 × 250 nm^2 square deposits of **6** at 100 °C, pitch 10 nm, dwell 500 µs, 5 kV, 2000 passes, 600 pA, top down and 50° tilt. A round, very granular halo is visible.

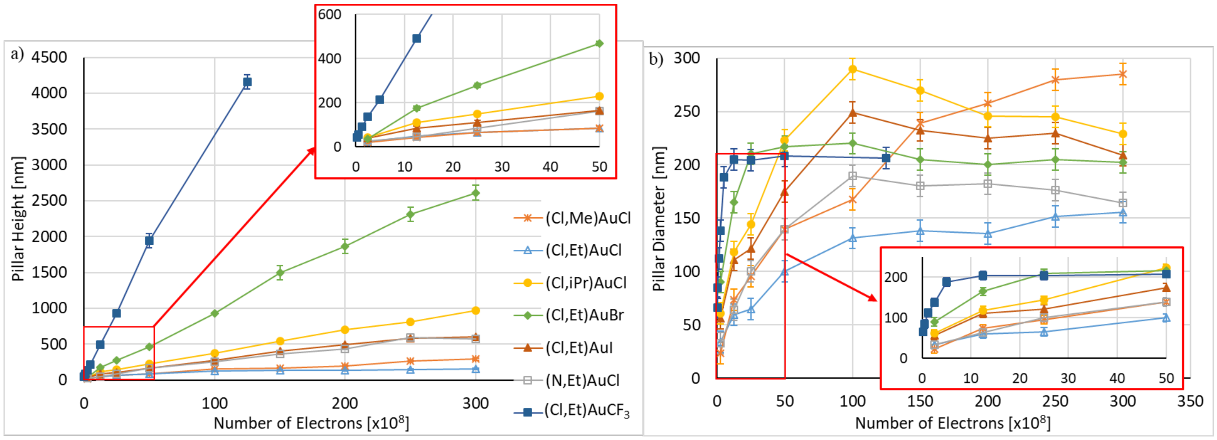

Figure S20: (a) Height and (b) diameter of pillars grown using a 5 kV, 40 pA beam as a function of the electron dose given as total number of primary electrons used to deposit a pillar. During all experiments the substrate and the precursor are heated together to 100 °C, except for (Cl,Me)AuCl, which was heated to 120 °C. For each precursor an array of 3 × 3 pillars was deposited. The lines between the points merely serve as a guide to the guide.
